# Light-Induced, Lysine-Targeting Irreversible Covalent Inhibition of the Human Oxygen Sensing Hydroxylase Factor Inhibiting HIF (FIH)

**DOI:** 10.1021/jacs.5c01935

**Published:** 2025-05-09

**Authors:** Yue Wu, Zhihong Li, Samanpreet Kaur, Zewei Zhang, Jie Yue, Anthony Tumber, Haoshu Zhang, Zhe Song, Peiyao Yang, Ying Dong, Fulai Yang, Xiang Li, Christopher J. Schofield, Xiaojin Zhang

**Affiliations:** † State Key Laboratory of Natural Medicines, Jiangsu Key Laboratory of Drug Design and Optimization, and Department of Chemistry, 56651 China Pharmaceutical University, Nanjing 211198, China; ‡ Chemistry Research Laboratory and the Ineos Oxford Institute for Antimicrobial Research, 6396 University of Oxford, 12 Mansfield Road, Oxford OX1 3TA, U.K.

## Abstract

Factor inhibiting hypoxia-inducible factor (FIH) is a JmjC domain 2-oxoglutarate (2OG) and Fe­(II)-dependent oxygenase that catalyzes protein hydroxylations, including of specific asparagines in the *C*-terminal transcriptional activation domains of hypoxia-inducible factor alpha (HIF-α) isoforms. FIH is of medicinal interest due to its ability to alter metabolism and modulate the course of the HIF-mediated hypoxic response. We report the development of a light-induced, lysine (Lys106)-targeting irreversible covalent inhibitor of FIH. The approach is complementary to optogenetic methods for regulation of transcription. The covalently reacting inhibitor **NBA-ZG-2291** was the result of structure-guided modification of the reported active site binding FIH inhibitor **ZG-2291** with an appropriately positioned *o*-nitrobenzyl alcohol (*o*-NBA) group. The results demonstrate that **NBA-ZG-2291** forms a stable covalent bond in a light-dependent process with Lys106 of FIH, inactivating its hydroxylation activity and resulting in sustained upregulation of FIH-dependent HIF target genes. The light-controlled inhibitors targeting a lysine residue enable light and spatiotemporal control of FIH activity in a manner useful for dissecting the context-dependent physiological roles of FIH.

## Graphical abstract

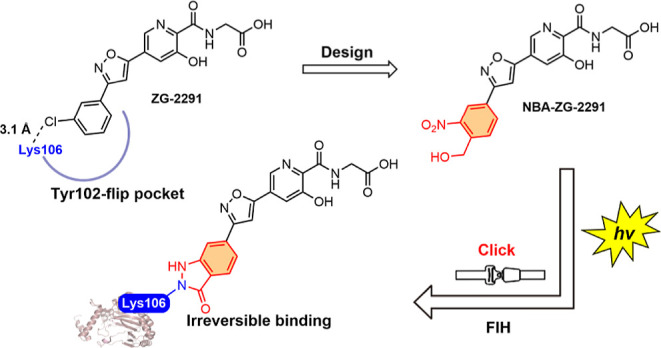

## Introduction

Oxygen is critical for cellular metabolism in all aerobic organisms, importantly by enabling oxidative phosphorylation in mitochondria, a process essential for efficient ATP generation.^1^ Cellular oxygen homeostasis is a fundamental physiological mechanism that regulates the balance between oxygen supply and consumption.^1^ Disruptions to this balance can lead to metabolic dysregulation and cellular dysfunction, highlighting the importance of robust oxygen/hypoxia sensing mechanisms and adaptive responses in response to variations in oxygen availability.^1^ In animals, the α,β-hypoxia-inducible factor (HIF) transcription factor is part of a regulatory system that controls many cellular responses to changes in oxygen availability, playing a pivotal role in various physiological processes and diseases, including the response to ischemia, tumorigenesis, anemia, and metabolic dysfunction.^2,3^

The stability of HIF-α, but not HIF-β, isoforms increases in hypoxia, as does the transcriptional activity of α,β-HIF, due to decreased catalysis by specific 2-oxoglutarate (2OG) and Fe­(II) dependent oxygenases, which are proposed to act as oxygen/hypoxia sensors.^4,5^ Under normoxic conditions, HIF-α isoforms are hydroxylated by prolyl hydroxylase domain (PHD) oxygenases;^6,7^ the resultant prolyl-hydroxylated HIF-α proteins bind strongly to the von Hippel-Lindau (VHL) E3 ligase complex, leading to ubiquitin-mediated proteasomal degradation, negatively regulating the stability of HIF-α.^8^ Inhibitors of the PHDs are used for anemia treatment via stabilization of HIF-2α, increasing expression of the erythropoietin (EPO) gene.^6^ To date, six PHD inhibitors have been approved for the treatment of anemia in chronic kidney disease.^9–11^ The activities of HIF isoforms are also influenced by another 2OG oxygenase—factor inhibiting HIF (FIH).^12^ FIH specifically hydroxylates asparagine residues within the *C*-terminal transactivation domains of HIF-1α and HIF-2α, preventing their interaction with the transcriptional coactivators/histone acetyl transferases p300/CBP, thus inhibiting transcriptional activation of HIF.^13^

In contrast to PHDs, which regulate HIF-α stability, FIH appears to fine-tune HIF activity by modulating its transcriptional potential. FIH also has multiple other non-HIF substrates, many from the ankyrin repeat domain family.^14,15^ The overall evidence suggests that FIH plays a key role in the cellular adaptation to hypoxia but also has roles in regulating metabolism in normoxia.^15^ Indeed, genetic deletion studies of FIH have not as yet reproduced the erythropoietic effects observed with PHD inhibitors.^16,17^ Genetic studies show that FIH deletion/reduction in mice causes an overall increase in oxygen consumption by upregulation of both oxidative metabolism and glycolysis. Significant decreases in body weight and reduced hepatic steatosis were observed in mice lacking FIH fed with a high-fat diet.^16,17^ Studies with small molecules have also demonstrated the potential of FIH inhibition to alter cellular metabolism.^18,19^

We have reported on a selective and reversibly binding FIH inhibitor,^18,19^ **ZG-2291**, binding of which causes a substantial change (flip) in the conformation of Tyr102 at the FIH active site (PDB ID: 8II0 ^18^), as also observed on binding of some 2OG derivatives to FIH.^20^ Importantly for the work described here, the rigidly linked phenyl ring of **ZG-2291** is bound tightly within this Tyr102-flip pocket, meaning the *meta*-chloro substituent of **ZG-2291** is positioned to interact with the *N* ^ε^ amino group of Lys106 (Figure 1A). Electron density for Lys106 has been rarely observed in other FIH structures (Figure S1), likely due to the high flexibility of its side chain in the absence of stabilizing interactions.^18^

**Figure 1:**
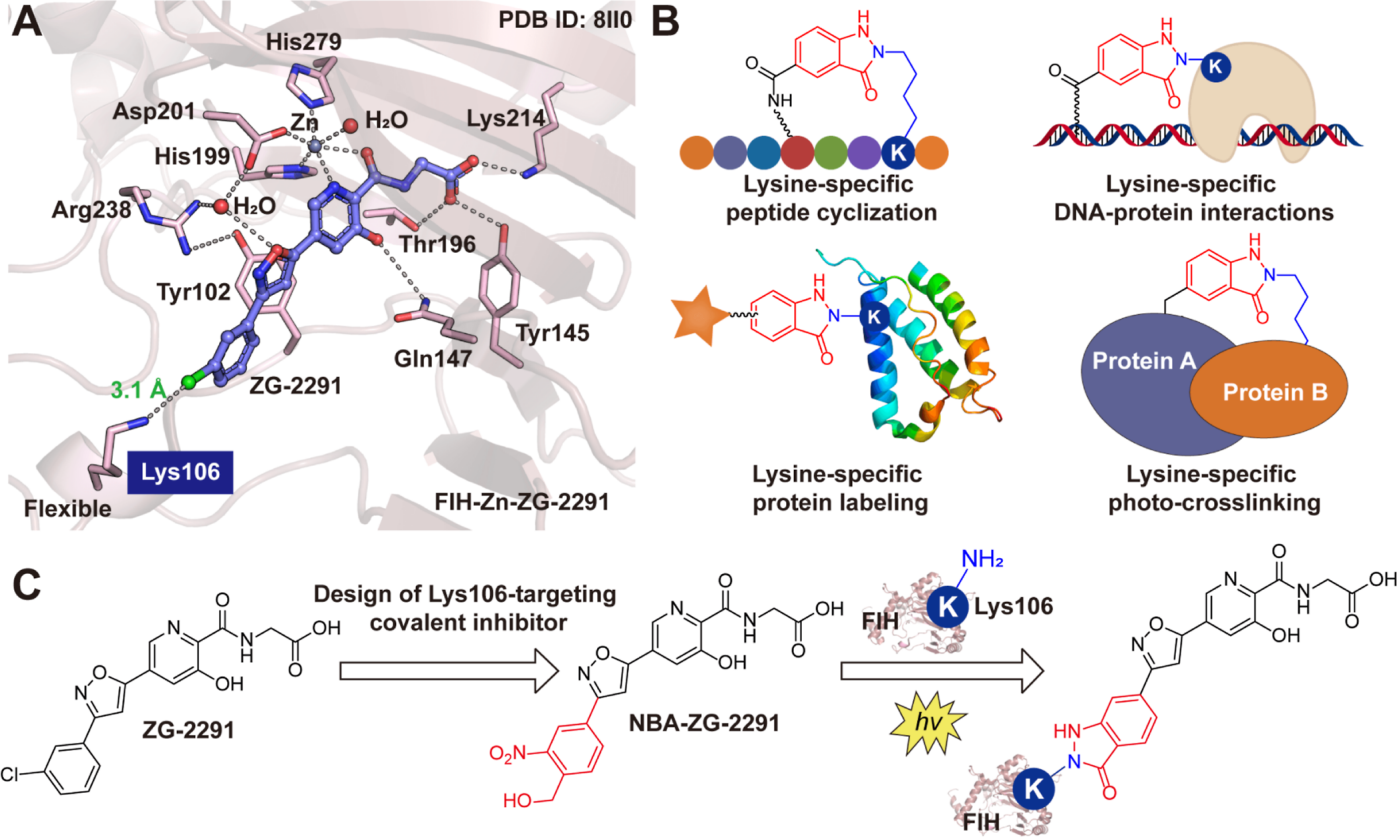
(A) View from a crystal structure of FIH in complex with the FIH inhibitor **ZG-2291** (PDB ID: 8II0 ^18^). Note that the interaction between the **ZG-2291** *meta*-chloro group and Lys106 enables electron density for the Lys106 side chain to be clearly observed, which has been rarely observed in reported FIH structures.^18,20,31–33^ (B) Previous applications of light-induced primary amines and the *o*-NBAs click cyclization reaction; the reaction proceeds via a nitroso intermediate (Figure S3). (C) Design of the light-induced, Lys106-targeting irreversible FIH covalent inhibitor, **NBA-ZG-2291**.

In part because HIF itself is highly inducible (by hypoxia), the HIF system is an excellent model system for developing a spatiotemporal understanding of epigenetic processes and other factors that regulate transcription. We are interested in understanding the factors that regulate the expression of sets of (potential) HIF target genes in different contexts, in particular with respect to the HIF hydroxylases.^21^ In this regard, small molecule inhibitors are of interest, since they might be used to upregulate specific sets of potential HIF target genes for treatment of diseases, including cancer.^22^

To date, most HIF hydroxylase inhibitors bind reversibly at the active sites.^15,23^ For biological studies, inhibitors that bind irreversibly and covalently, including those where covalent cross-linking can be initiated by light, are of particular interest for studying time-dependent effects on transcription. While there have been considerable efforts to develop “optogenetic” methods for the regulation of transcription,^24,25^ including for the CREB transcription factor,^26^ less work has been done on light-activated small molecule regulators of transcription.

Inspired by the structural observations with **ZG-2291**, we proposed the design of covalently reacting FIH inhibitors that leverage the position and flexibility of Lys106 (Figure 1A). We envisaged the identification of an appropriate electrophilic warhead capable of selectively reacting with the *N* ^ε^ amino group of Lys106 in a light-induced manner.

A light-induced cyclization click reaction between primary amines and *o*-nitrobenzyl alcohols (*o*-NBAs) has been reported for the modular functionalization of a wide range of small molecules and native proteins.^27–29^ This approach has been applied to lysine-specific peptide cyclization, lysine-specific protein labeling and covalently capturing protein–protein/DNA interactions in living cells^27–30^ (Figure 1B). Here, we describe the development of FIH binding scaffolds that enable the light-induced covalent modification of Lys106 of FIH (Figure 1C). We demonstrate that **NBA-ZG-2291** covalently reacts with FIH upon activation by light causing efficient inactivation of FIH-catalyzed hydroxylation so enabling controlled studies on the roles of FIH in oxygen-sensing and other pathways.

## Results and Discussion

To explore the possibilities of covalently targeting FIH, we examined a cocrystal structure of **ZG-2291** and FIH (PDB ID: 8II0 ^18^); the structures reveal that **ZG-2291** binds at the FIH active site and coordinates to the active site metal ion via its pyridine nitrogen and glycinamide oxygen. The side chain carboxylate of **ZG-2291** is positioned to form electrostatic/hydrogen bonding interactions with the side chains of the active site residues Tyr145 and Lys214. A substantial change in the side chain conformation of Tyr102 occurs forming a Tyr102-flipped pocket. The rigidly linked phenyl ring of **ZG-2291** is bound tightly within the Tyr102-flip pocket, formed by Tyr102, Tyr103, Asp104, Glu105, and Lys106. Notably, the *meta*-chloro substituent of **ZG-2291** is positioned to interact with the *N* ^ε^ amino group of Lys106 (Figure 1A), electron density for which has been rarely observed in other FIH structures (Figure S1) due to the high flexibility of its side chain.^18,20,31–33^ The *meta*-chloro moiety in **ZG-2291** is ∼3.1 Å from the *N* ^ε^ amino group of Lys106 in its crystalline complex with FIH (Figure 1A). Inspired by the unique positioning of Lys106, we proposed a covalently reacting inhibitor capable of selectively reacting with Lys106 upon light activation, by incorporating a light-sensitive *o*-NBA group into **ZG-2291** scaffold (Figure 1C). This mechanism may provide spatiotemporal control over FIH inhibition, a significant advantage over traditional reversibly binding FIH inhibitors.

To investigate the feasibility of the covalent inhibitor design, we first investigated the reactivity of **NBA-ZG-2291** with Cbz-Lys-OMe, i.e. a diprotected lysine residue, under 365 nm light-mediated activation in the buffer (Figure 2A). Liquid chromatography–mass spectrometry (LC–MS) experiments were conducted to monitor the formation of the potential indazolone product. The reaction proceeded smoothly upon light activation (365 nm, 16 W). As the irradiation time increased, levels of the **NBA-ZG-2291** reactant gradually decreased, while the product levels gradually increased (Figure 2B). The product was identified as **3** by mass spectrometric analysis (Figure S2). After 2 h of light exposure, the reaction reached saturation with a yield of ∼90% (Figure 2C). **NBA-ZG-2291** and Cbz-Lys-OMe in the buffer can undergo an aldehyde-amine ligation-like coupling reaction under UV irradiation. The reaction begins with the photolysis of *o*-NBA rapidly generating *o*-nitroso benzaldehyde. The primary amine of Cbz-Lys-OMe undergoes condensation with *o*-nitroso benzaldehyde, leading to subsequent formation of the indazolone product (Figure S3).^34^

**Figure 2:**
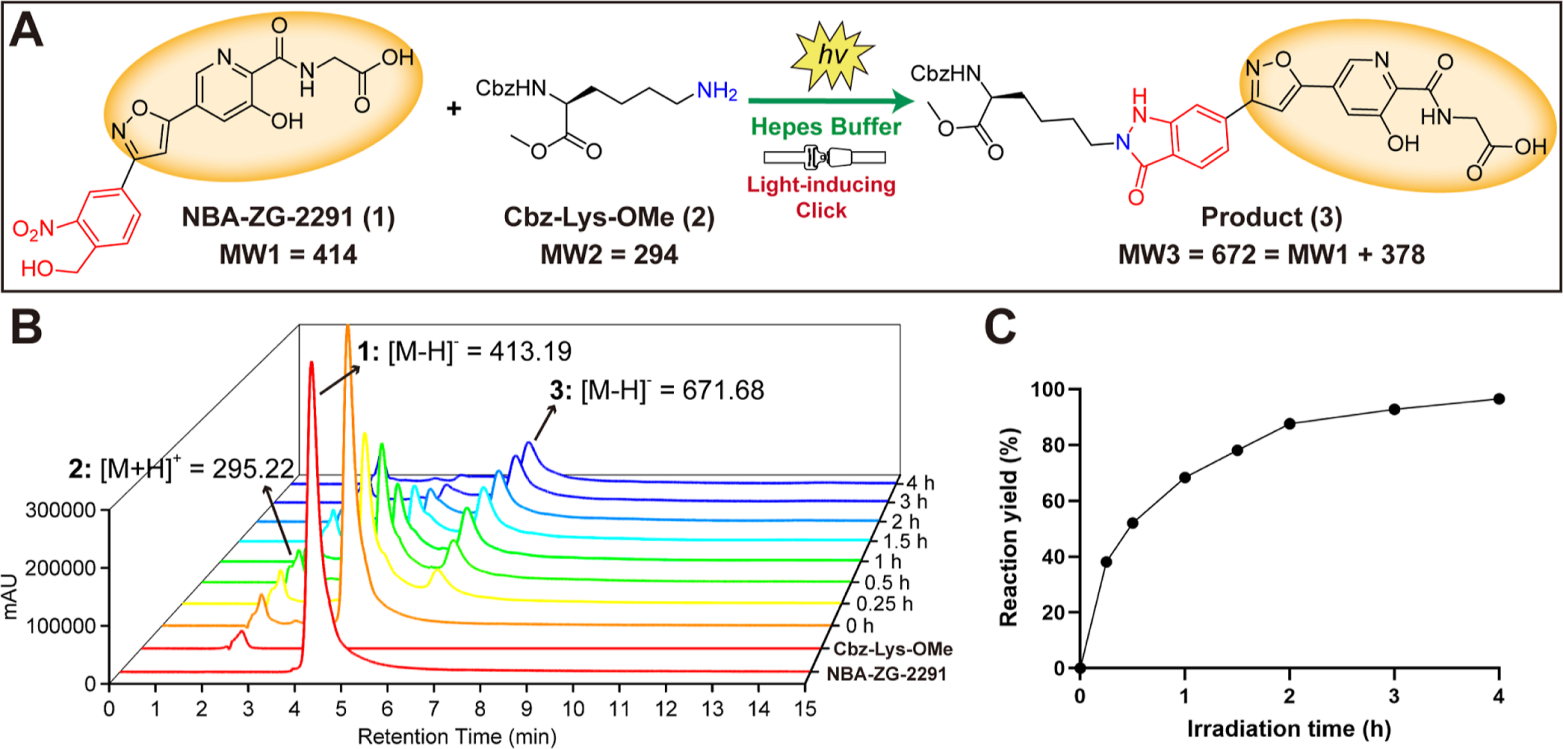
Light-induced click conjugation between **NBA-ZG-2291** (500 μM) and Cbz-Lys-OMe (lysine residue, 2 mM). (A) Light-induced click conjugation of primary amine **2** with *o*-NBA derivate **1**. (B) Peaks for the conjugated product (**3**) increased after light activation. (C) The reaction proceeded efficiently after light activation.

Based on the proposed mechanism, we designed and synthesized two control compounds lacking the *para*-hydroxy methyl group (**Neg-1**, Table S1, Figure 4A) or the *meta*-nitro group (**Neg-2**, Table S2, Figure 4A) compared to **NBA-ZG-2291**. The LC–MS results suggest that under light activation, neither of the control compounds undergoes click reaction with Cbz-Lys-OMe (Tables S1 and S2).

Furthermore, we crystallized **NBA-ZG-2291** in complex with FIH without irradiation, using Mn­(II) as a stable surrogate for Fe­(II) (Figure 3B). The resultant structure reveals that **NBA-ZG-2291** binds at the FIH active site and coordinates with the active site metal ion. Attempts to crystallize **NBA-ZG-2291** with FIH upon light activation were, however, unsuccessful in producing a cross-linked complex. Analysis of the crystal structure of FIH in complex with **NBA-ZG-2291** compared to that with **ZG-2291** implies that the introduction of the *o*-NBA group prevents the side chain phenyl group from fitting into the Tyr102-flip pocket, leading to a reversal of the isoxazole ring conformation in **NBA-ZG-2291** compared to **ZG-2291** (Figure 3C). The isoxazole ring in **NBA-ZG-2291** is not positioned to interact with Arg238 and does not induce the conformational flip of Tyr102 (Figures 3C and S4). The lack of interaction of **ZG-2291** with Lys106 in the crystalline state fails to constrain its flexible conformation, highlighting the importance of both noncovalent and covalent interactions with Lys106.

**Figure 3:**
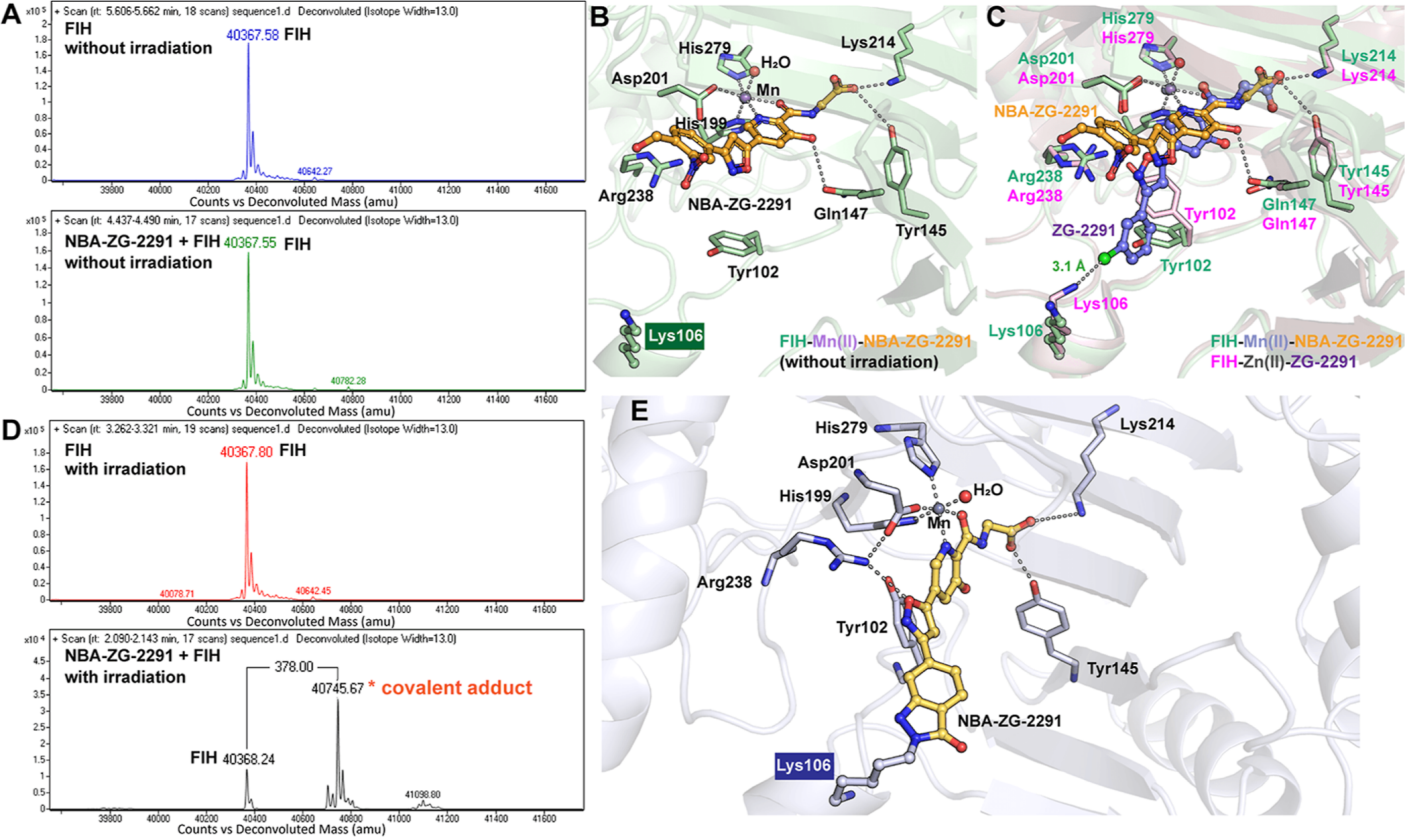
(A) Intact protein mass analysis of FIH (5 μM) and FIH (5 μM) treated with **NBA-ZG-2291** (50 μM) without irradiation. (B) Crystal structure view of FIH in complex with **NBA-ZG-2291** without irradiation. (C) Superimposition of the crystal structure FIH in complex with **NBA-ZG-2291** (green) (PDB ID: 9I4H) and that in complex with **ZG-2291** (pink) (PDB ID: 8II0 ^18^). (D) Intact protein mass analysis of FIH (5 μM) and FIH (5 μM) treated with **NBA-ZG-2291** (50 μM) followed by 30 min of irradiation (365 nm, 16 W). The replicates for intact protein mass analysis are shown in Figure S10. (E) Predicted covalent binding mode of **NBA-ZG-2291** with FIH.

Subsequently, we verified the covalent reaction of **NBA-ZG-2291** and FIH with irradiation in solution using, intact protein mass analyzes. The intact protein MS analysis shows that, after 30 min of light activation (365 nm, 16 W), a new peak with the anticipated mass shift corresponding to covalent reaction was detected, with a high extent of modification (Figure 3D). As expected, intact protein mass spectrometric (MS) analysis failed to detect the covalent adducts (Figure 3A) in the absence of irradiation.

The results of dihedral angle scan analyzes indicate that the energy barrier associated with the single bond rotation between the pyridine and isoxazole rings is below 5 kcal/mol, suggesting that the rotation around this bond is energetically feasible (Figure S4B). This conformational flexibility may enable the covalent warhead, *o*-NBA, to adopt an appropriate orientation for cross-linking, facilitating its covalent reaction with Lys106.

To obtain insight into the covalent binding mode of FIH with **NBA-ZG-2291**, **NBA-ZG-2291** modified FIH Lys106 was customized as nonstandard residue and the **NBA-ZG-2291**-FIH complex was prepared to conduct 100 ns unrestricted molecular dynamics (MD) simulations. The root-mean-square deviation (RMSD) of the complex quickly reached equilibrium after 10 ns and the RMSD converged to 3.0 Å (Figure S4A). Furthermore, the dihedral angle of the isoxazole-5-ylpyridine fragment in **NBA-ZG-2291** converged to 30° (Figure S5B), with the oxygen of isoxazole faces Arg238 (Figure S4B), forming a stable interaction between the oxygen of the isoxazole and Arg238 (Figure 3E and Movie S1). We also analyzed the root-mean-square fluctuation (RMSF) of each residue to evaluate their flexibility during the simulation. Notably, **NBA-ZG-2291**-modified Lys106 exhibited a significantly low RMSF (Figure S5B). This indicates that the covalent interaction at Lys106 substantially reduces its conformational flexibility, suggesting a stable binding interaction. Such stability is consistent with the expected behavior of a covalent inhibitor, where the covalent bond formation typically limits the movement of the involved residue. These findings further demonstrated the stability of the covalent binding between FIH and **NBA-ZG-2291** (Figure 3E).

We also performed a reported affinity-based fluorescence polarization (FP) assay (Figure S6)^35,36^ under light activation to measure the binding affinity of **NBA-ZG-2291** to FIH (Figure 4A). In the absence of irradiation, **NBA-ZG-2291** exhibits a weak binding affinity to FIH^*WT*^, with a *K*_i_ value of 423 nM (Figure 4B). As the irradiation time was extended, the binding affinity of **NBA-ZG-2291** to FIH progressively strengthened, suggesting the progressive formation of new interactions. After 30 min of irradiation, the binding affinity stabilized, with a *K*_i_ of approximately 40 nM determined (Figures 4B and S7), indicating that the covalent reaction had reached near equilibrium. These observations are consistent with the intact protein MS analyzes. We also evaluated the binding affinities of two negative controls to FIH, which were designed not to covalently react with Lys106. The results demonstrate that the binding affinities of these control compounds to FIH were unaffected by irradiation (Figures 4B and S7), suggesting that the light-induced interaction between **NBA-ZG-2291** and FIH arises from the covalent reaction of the *o*-NBA group of **NBA-ZG-2291** with Lys106.

**Figure 4:**
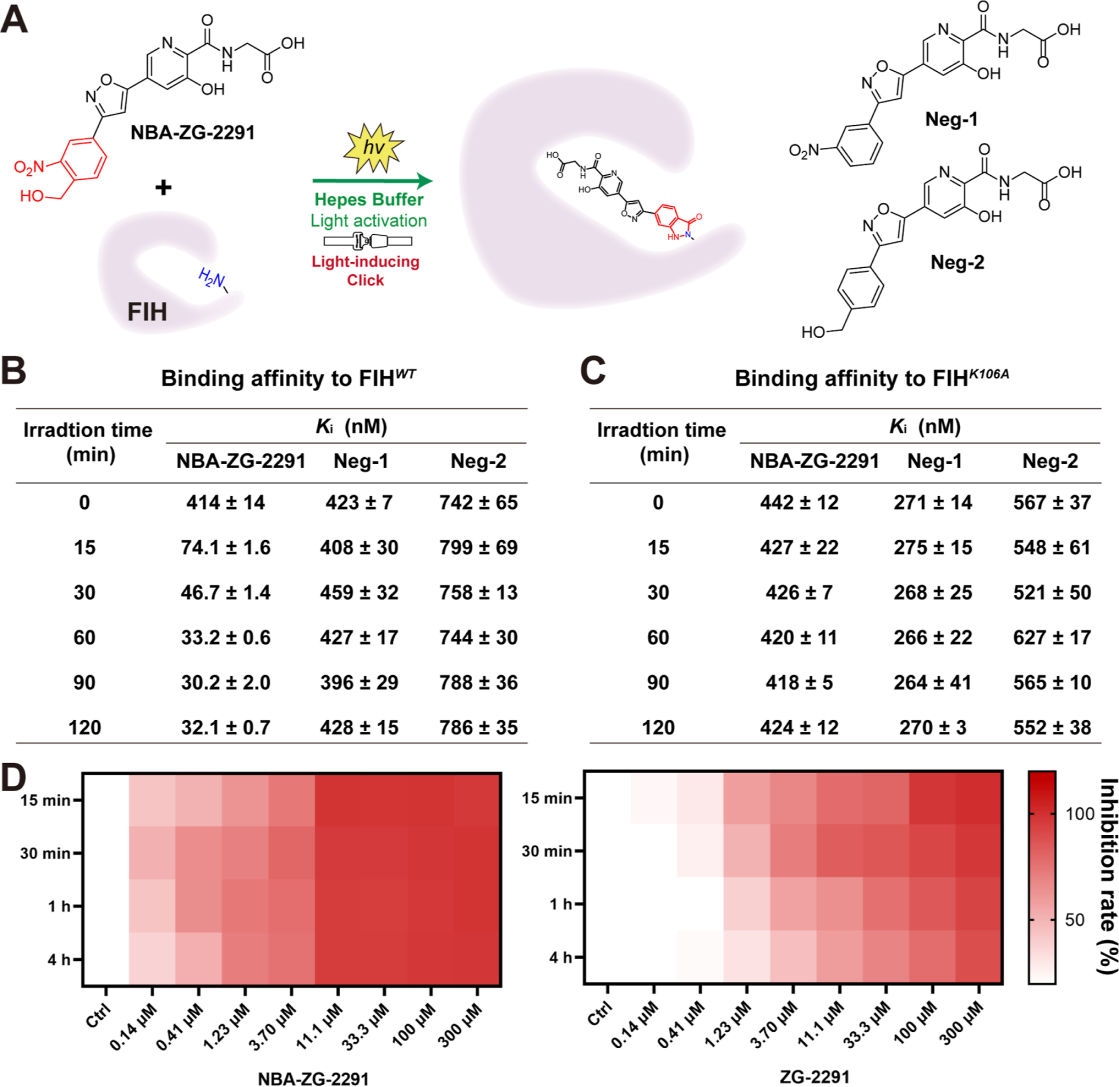
(A) Light-induced click conjugation between **NBA-ZG-2291** and FIH protein. (B) Light-induced binding affinity of **NBA-ZG-2291** to FIH^*WT*^. (C) Light-induced binding affinity of **NBA-ZG-2291** to FIH^*K106A*^. (D) **NBA-ZG-2291** exhibited long-term inhibition of the FIH-catalyzed hydroxylation reaction.

To further verify the covalent binding between **NBA-ZG-2291** and Lys106, the FIH^*K106A*^ variant was produced and purified. FP assay results indicated that the binding affinity of **NBA-ZG-2291** for the FIH^*K106A*^ variant remained unchanged over varying irradiation times (Figures 4C and S8), further confirming the covalent interaction with Lys106.

To explore whether the covalent inhibitor can effectively and sustainably prevent the hydroxylation process catalyzed by FIH, the inhibitory activities against FIH were evaluated using the fluorescent *O*-phenylenediamine (OPD) 2OG derivatization turnover assay (Figure S9).^37^ The results show that **NBA-ZG-2291** effectively reduces the consumption of 2OG (Table S3), indicating inhibition of the hydroxylation activity of FIH on the HIF peptide (Figure 4D). Compared to **ZG-2291** (Figure 4D and Table S4), **NBA-ZG-2291** demonstrates more potent and sustained inhibition of FIH hydroxylation activity.

To investigate potential cellular target engagement of **NBA-ZG-2291** with FIH, we performed a time-dependent cellular thermal shift assay (CETSA) using Hep3B cells. The results imply that, after 30 min of light activation (365 nm, 16 W), **NBA-ZG-2291** effectively stabilizes FIH with a Δ*T*_m_ of about 9 °C after incubation for 12–48 h (Figure 5A), indicating sustained engagement with FIH. By contrast, **NBA-ZG-2291** was not observed to affect the stability of PHD2 (Figure S11) in cells at the tested concentration, implying that **NBA-ZG-2291** selectively engages with FIH in cells. We also performed Western blotting to examine the protein levels of both HIF-1α and HIF-2α in control and **NBA-ZG-2291**-treated Hep3B cells. The results demonstrate that **NBA-ZG-2291** did not significantly alter the levels of either HIF-1α or HIF-2α, further supporting the proposal that **NBA-ZG-2291** does not substantially interfere with PHD activity (Figure S12).

**Figure 5:**
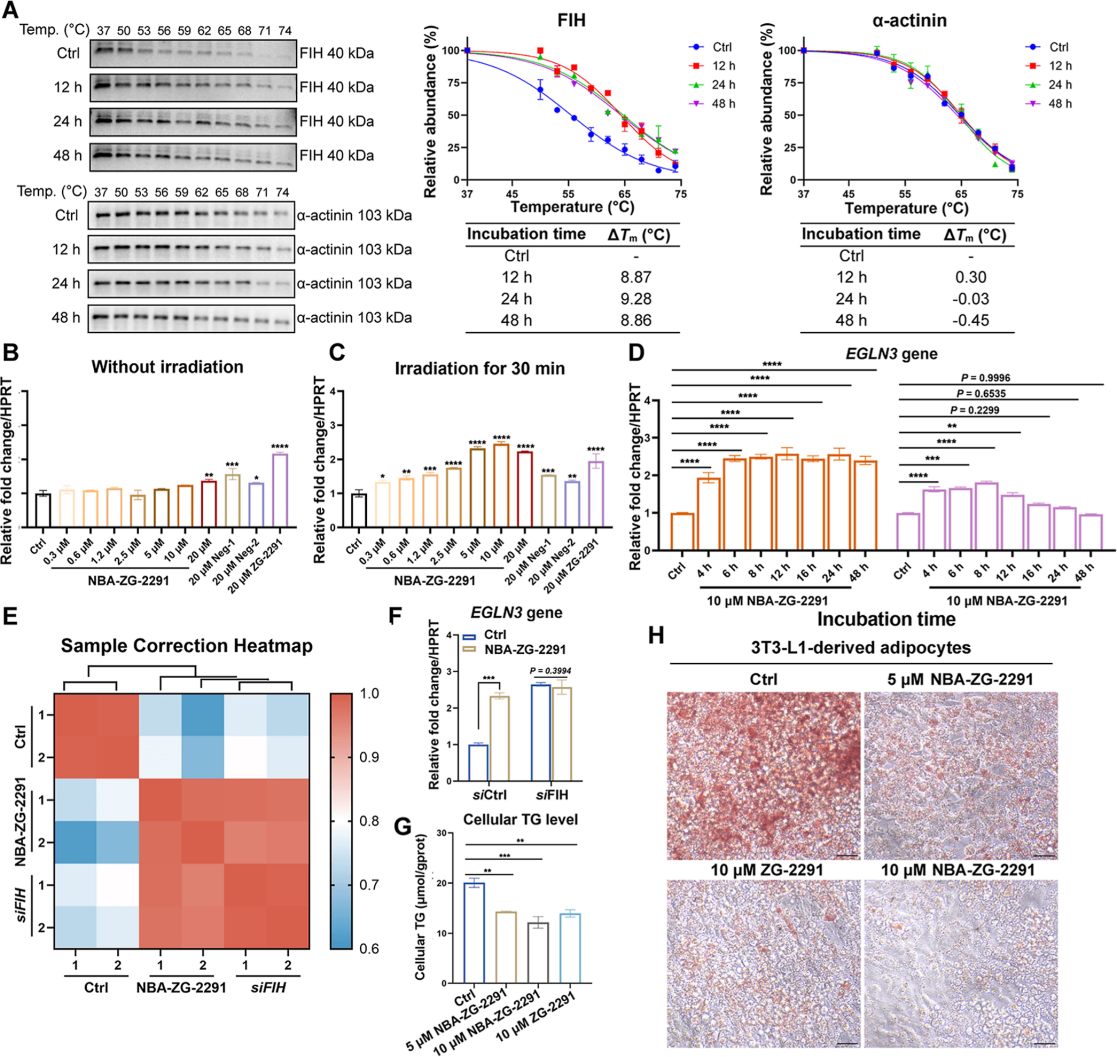
**NBA-ZG-2991** sustainably engages with FIH and regulates the expression of FIH-dependent HIF target gene *EGLN3* in Hep3B cells. (A) The CETSA assay was carried out with Hep3B cells grown in the presence of **NBA-ZG-2291** (50 μM) for 12–48 h after 30 min of light activation (365 nm, 16 W). The intensity of the bands for grayscale analysis was referenced to the starting temperature (37 °C). α-Actinin was the sample loading control. *n* = 2; mean ± SD. The uncropped blot is shown in Figure S14. (B) **NBA-ZG-2291** showed weak upregulation to the expression of *EGLN3* without irradiation. (C) **NBA-ZG-2291** showed significant and dose-dependent upregulation of the expression of *EGLN3* with irradiation. (D) **NBA-ZG-2291** showed long-term regulation of expression of the FIH-dependent HIF target gene after 30 min of irradiation. (E) Correlation heatmap of the **NBA-ZG-2291**-treated group and the siFIH group by RNA-sequencing analysis. (F) **NBA-ZG-2291** showed no significant effect on *EGLN3* expression when FIH was knocked down. The relative fold change with respect to DMSO treated sample is shown (with normalization to HPRT gene levels). Mean ± SEM; *n* = 3. (G) TG level of **NBA-ZG-2291**-treated 3T3-L1-derived adipocytes. Mean ± SEM; *n* = 3. (H) Representative Oil Red O-stained images of the control, **ZG-2291**-treated, and **NBA-ZG-2291**-treated 3T3-L1-derived adipocytes. Scale Bar, 30 μm. P values were analyzed by two-way ANOVA comparing with the vehicle/control group (*, *P* < 0.05; **, *P* < 0.01; ***, *P* < 0.001, ****, *P* < 0.0001). See Methods (Quantitative PCR Analysis of Gene Expression) for assay details.

The Egl-9 family hypoxia-inducible factor 3 gene (*EGLN3*, *PHD3*) is a HIF target gene, expression of which is negatively regulated by FIH catalysis,^21,38^ potentially in a context-dependent manner. We therefore investigated the effect of the light-induced covalent inhibitor **NBA-ZG-2291** on *EGLN3* expression levels in Hep3B cells using **ZG-2291** as a positive inhibition control. Quantitative real-time PCR (qRT-PCR) analysis revealed that, in the absence of irradiation, **NBA-ZG-2291** showed weak upregulation of *EGLN3* mRNA level. Treatment of Hep3B cells with 10 μM or 20 μM **NBA-ZG-2291** did not result in significant upregulation and very weak upregulation, respectively, of the expression of *EGLN3* in the absence of irradiation, while **ZG-2291** (20 μM) clearly upregulated the *EGLN3* mRNA level by ∼2.2-fold. By contrast, in the presence of irradiation **NBA-ZG-2291** significantly upregulates *EGLN3* mRNA levels in a dose-dependent manner. After 30 min of light activation (365 nm, 16 W), treatment of Hep3B cells with a relatively low concentration of **NBA-ZG-2291** (0.3 μM) resulted in clear (1.35-fold) upregulation of *EGLN3*. At a concentration of 10 μM, **NBA-ZG-2291** increased *EGLN3* expression by ∼2.5-fold with irradiation. The expression of the FIH-dependent *EGLN3* gene was not affected by irradiation with the two negative control compounds or **ZG-2291** (Figure 5C).

We also compared the time-dependent regulation of **NBA-ZG-2291** on the HIF pathway with that of **ZG-2291**. Within 6 h, **NBA-ZG-2291** upregulated *EGLN3* expression in a time-dependent manner, maintaining a 2.5-fold increase from 6 to 48 h (Figure 5D). In contrast, although **ZG-2291** time-dependently upregulates *EGLN3* expression within 8 h, after 12 h, the mRNA levels of *EGLN3* were observed to gradually decrease (Figure 5D). After 16 h treatment with **ZG-2291**, the *EGLN3* mRNA levels in **ZG-2291**-treated Hep3B cells showed no significant difference compared to the negative control (DMSO). This may result from the limited action time of the reversible inhibitor, perhaps due to its metabolism. The overall results provide strong evidence that the light-induced, lysine-targeting irreversible covalent inhibitor **NBA-ZG-2291** can effectively and sustainably engage FIH, leading to the upregulation of FIH-dependent HIF target gene expression.

To confirm whether the effects of **NBA-ZG-2291** on gene expression are FIH-dependent, we knocked down FIH using siRNA and compared the impact of **NBA-ZG-2291** treatment and FIH knockdown (siFIH) on gene expression in Hep3B cells. Whole-genome expression profiling analysis was conducted in **NBA-ZG-2291**-treated Hep3B cells and FIH knockdown Hep3B cells, with DMSO-treated Hep3B cells serving as controls. The heatmap displays the expression patterns of differentially expressed genes (DEGs) across the three experimental groups (Figure S13). The **NBA-ZG-2291**-treated and siFIH groups exhibit a high degree of similarity, indicating that **NBA-ZG-2291** exerts its effects primarily through FIH inhibition. Differences between these two groups might arise from the fact that **NBA-ZG-2291** specifically inhibits the enzymatic activity of FIH, whereas FIH knockdown may also affect its nonenzymatic/structural functions. Furthermore, sample correlation analysis revealed a high similarity in gene expression between the **NBA-ZG-2291**-treated group and the siFIH group, as shown by the correlation heatmap (Figure 5E). This finding further indicates that the effects of **NBA-ZG-2291** are FIH-dependent. Additionally, PCR results showed that **NBA-ZG-2291** had no significant effect on *EGLN3* expression when FIH was knocked down (Figure 5F), further supporting its specificity for FIH.

Previous studies have indicated that FIH knockout or inhibition has potential to alleviate metabolic diseases such as obesity and fatty liver diseases.^17,18^ Therefore, we investigated the effect of **NBA-ZG-2291** on lipid homeostasis in 3T3-L1-derived adipocytes. Treatment with **NBA-ZG-2291** significantly improved cellular triglyceride levels (Figure 5G). Oil Red O staining showed that **NBA-ZG-2291**-treated adipocytes manifest reduced lipid droplet accumulation compared with control cells (Figure 5F). These findings highlight **NBA-ZG-2291** as a light-induced covalent FIH inhibitor with potential metabolic regulatory effects, contributing to improved cellular lipid homeostasis.

## Discussion

FIH plays a crucial role in regulating cellular responses to oxygen availability in animals by hydroxylating HIF-α subunits, thereby fine-tuning HIF transcriptional activity.^13^ In previous work, we developed **ZG-2291**, a potent and selective FIH inhibitor that induced substantial conformational change of Tyr102 in FIH active site.^18^ Notably, the *meta*-chloro substituent of **ZG-2291** is positioned to interact with the *N* ^ε^ amino group of Lys106, whose electron density is rarely observed in other FIH structures. In this study, we developed **NBA-ZG-2291**, a light-induced, irreversible covalent inhibitor of FIH, which leverages the flexibility of Lys106 in the Tyr102-flip pocket. The introduction of an *o*-NBA moiety to the terminal phenyl group of **ZG-2291** enabled the discovery of **NBA-ZG-2291**, which covalently reacts with Lys106 upon UV light activation, providing a spatiotemporally controlled inhibition mechanism. Biophysical studies provide evidence that **NBA-ZG-2291** binds at the FIH active site and covalently reacts with Lys106 upon light activation.

Our study demonstrates that **NBA-ZG-2291** not only manifests potent and sustained inhibition of FIH but enables modulation of FIH activity over extended periods. **NBA-ZG-2291** effectively upregulates an FIH-dependent HIF target gene^21,38^ in an irradiation-dependent manner. The comparison with the FIH knockdown group underscores the specificity of **NBA-ZG-2291** for FIH, as a high correlation in gene expression was observed. These findings highlight the potential of **NBA-ZG-2291** as a powerful tool for studying FIH-dependent cellular processes, in particular as a light-controlled spatiotemporal probe for dissecting the context-dependent physiological roles of FIH over a variety of time scales. Furthermore, our results provide compelling evidence for the metabolic regulatory potential of FIH, because **NBA-ZG-2291** significantly improves lipid homeostasis in adipocytes.

Covalently reacting FIH inhibitors have distinct advantages over noncovalent inhibitors, including prolonged target engagement and enhanced therapeutic efficacy. The ability to form covalent bonds with key residue in the FIH active site may also reduce the risk of off-target effects, making it a promising strategy for time-based cellular studies on the roles of FIH in metabolic and other diseases, in a manner complementary to optogenetic and other covalent approaches for the regulation of transcription.^21,39^ The successful development of **NBA-ZG-2291** exemplifies the promise of light-induced, irreversible covalently reacting small molecule inhibitors as precision modulators enzyme function, with applications involving regulation of transcription extending well beyond FIH/the HIF system.

## Supplementary Material







## Notes

Whole-genome RNA sequencing (XLSX)
Animations of MD simulation of **NBA-ZG-2291**-FIH complex (MP4)
Original mass spectrometry, inhibition curves of FP assay, original data of OPD fluorescent derivative assay, molecular simulation results, experimental procedures, general chemistry procedures, synthesis schemes and methods, NMR spectra, and HR-MS spectra (PDF)

## Abbreviations

CETSA: cellular thermal shift assay
EGLN3: Egl-9 family hypoxia-inducible factor 3
EPO: erythropoietin
FIH: factor inhibiting hypoxia-inducible factor
HIF-α: hypoxia-inducible factor alpha
LC-MS: liquid chromatography–mass spectrometry
MD: molecular dynamics
MS: mass spectrometric
2OG: 2-oxoglutarate
*o*-NBA: *o*-nitrobenzyl alcohol
OPD: *O*-phenylenediamine
PHD: prolyl hydroxylase domain
qRT-PCR: quantitative real-time PCR
RMSD: root-mean-square deviation
RMSF: root-mean-square fluctuation
VHL: von Hippel-Lindau.
