# Supplementary material for: Light-Induced, Lysine-Targeting Irreversible Covalent Inhibition of the Human Oxygen Sensing Hydroxylase Factor Inhibiting HIF (FIH)
Source: J Am Chem Soc. 2025 May 9;147(21):17871–9. doi: 10.1021/jacs.5c01935 (PMC12123601; doi:10.1021/jacs.5c01935)
Supplement: Supplementary file 3 [file ja5c01935_si_003.pdf]

Supporting Information

Light-Induced, Lysine-Targeting Irreversible Covalent Inhibition of the Human Oxygen Sensing Hydroxylase Factor Inhibiting HIF (FIH)

Yue Wu,⋈,[a] Zhihong Li,⋈,[a] Samanpreet Kaur,⋈,[b] Zewei Zhang,⋈,[a] Jie Yue,[a] Anthony Tumber,[b] Haoshu Zhang,[a] Zhe Song,[a] Peiyao Yang,[a] Ying Dong,[a] Fulai Yang,[a] Xiang Li,[a] Christopher J. Schofield,*,[b] and Xiaojin Zhang*,[a]

[a] State Key Laboratory of Natural Medicines, Jiangsu Key Laboratory of Drug Design and Optimization, and Department of Chemistry, China Pharmaceutical University, Nanjing 211198 (China). E-mail: zxj@cpu.edu.cn

[b] Chemistry Research Laboratory and the Ineos Oxford Institute for Antimicrobial Research, University of Oxford, 12 Mansfield Road, Oxford OX1 3TA (United Kingdom). E-mail: christopher.schofield@chem.ox.ac.uk

⋈ These authors contributed equally.

KEYWORDS: factor inhibiting hypoxia inducible factor (FIH), 2-oxoglutarate dependent oxygenases, protein hydroxylation, regulation of transcription, lysine-targeting inhibition, covalent inhibitor, light-induced inhibition, oxygen / hypoxia sensing

ABSTRACT: Factor inhibiting hypoxia-inducible factor (FIH) is a JmjC domain 2-oxoglutarate (2OG) and Fe(II)-dependent oxygenase that catalyzes protein hydroxylations, including of specific asparagines in the *C*-terminal transcriptional activation domains of hypoxia-inducible factor alpha (HIF-α) isoforms. FIH is of medicinal interest due to its ability to alter metabolism and modulate the course of the HIF-mediated hypoxic response. We report the development of a light-induced, lysine (Lys106)-targeting irreversible covalent inhibition of FIH. The approach is complementary to optogenetic methods for the regulation of transcription. The covalently reacting inhibitor **NBA-ZG-2291** was the result of structure-guided modification of reported active site binding FIH inhibitor **ZG-2291** with an appropriately positioned *o*-nitrobenzyl alcohol (*o*-NBA) group. The results demonstrate that **NBA-ZG-2291** forms a stable, light-dependent covalent bond with Lys106 of FIH, inactivating its hydroxylation activity and resulting in sustained upregulation of FIH-dependent HIF target genes. The light-controlled inhibitors targeting a lysine residue enable light and spatiotemporally control of FIH activity in a manner useful for dissecting the context-dependent physiological roles of FIH.

Table of Contents

Table of Contents **S2**

1. Supplementary figures **S3**

Figure S1. Superimposition of FIH crystal structure derived views analyzing the conformation of Lys106 .... S3

Figure S2. Mass spectrometry of **NBA-ZG-2291** (**1**), Cbz-Lys-OMe (**2**), and the cross-linked product (**3**). S4

Figure S3. Proposed outline mechanism for the light-induced click reaction between **NBA-ZG-2291** (**1**) and Cbz-Lys-OMe (**2**) ... S5

Figure S4. Dihedral angle analysis for the isoxazole-5-yl pyridine fragment S6

Figure S5. Molecular dynamics (MD) simulations of **NBA-ZG-2291**-FIH complex S7

Figure S6. Schematic of the FP assay for FIH S8

Figure S7. Inhibition curves for **NBA-ZG-2291**, **Neg-1**, and **Neg-2** against FIH^{WT} under light activation S9

Figure S8. Inhibition curves of **NBA-ZG-2291**, **Neg-1**, and **Neg-2** against FIH^{K106A} under light activation S10

Figure S9. Schematic of the *O*-phenylenediamine (OPD) fluorescent derivative assay S11

Figure S10. Replicates for intact protein mass analysis S12

Figure S11. The CETSA assay detecting PHD2 engagement S13

Figure S12. Representative western blot visualization of HIF-1α and HIF-2α levels in Hep3B cells S14

Figure S13. Clustering heatmap of differentially expressed genes (DEGs) S15

Figure S14. Uncropped images of all western blots S16

2. Supplementary tables **S17**

Table S1. LC-MS Analysis of **Neg-1** and Cbz-Lys-OMe (**2**) Incubation in Hepes Buffer with Irradiation (365 nm, 16 W). S17

Table S2. LC-MS Analysis of **Neg-2** and Cbz-Lys-OMe (**2**) Incubation in Hepes Buffer with Irradiation (365 nm, 16 W). S18

Table S3. The Concentration of Remaining 2OG in the FIH Hydroxylation System after Incubation with **NBA-ZG-2291**. S19

Table S4. The Concentration of Remaining 2OG in the FIH Hydroxylation System after Incubation with **ZG-2291**. S20

Table S5. Data Collection and Refinement Statistics (Structures were Solved by Molecular Replacement). S21

3. Experimental procedures **S22**

3.1 Fluorescence polarization (FP) assays with FIH^{WT} and $FIHK^{106A}$ S22

3.2 *O*-Phenylenediamine (OPD) fluorescent derivative assay S22

3.3 X-ray crystallography S22

3.4 Intact protein mass spectrometry S23

3.5 Dihedral angle scan analysis S23

3.6 Molecular dynamics (MD) simulations S23

3.7 Cell culture S23

3.8 Cellular thermal shift assay (CETSA) S23

3.9 Quantitative PCR analysis of gene expression S24

3.10 siRNA-mediated knockdown of FIH S24

4. Synthesis **S24**

^{1}H NMR, HR-MS, and ^{13}C NMR spectra of A2-A3 and final products. S28

1. Supplementary figures

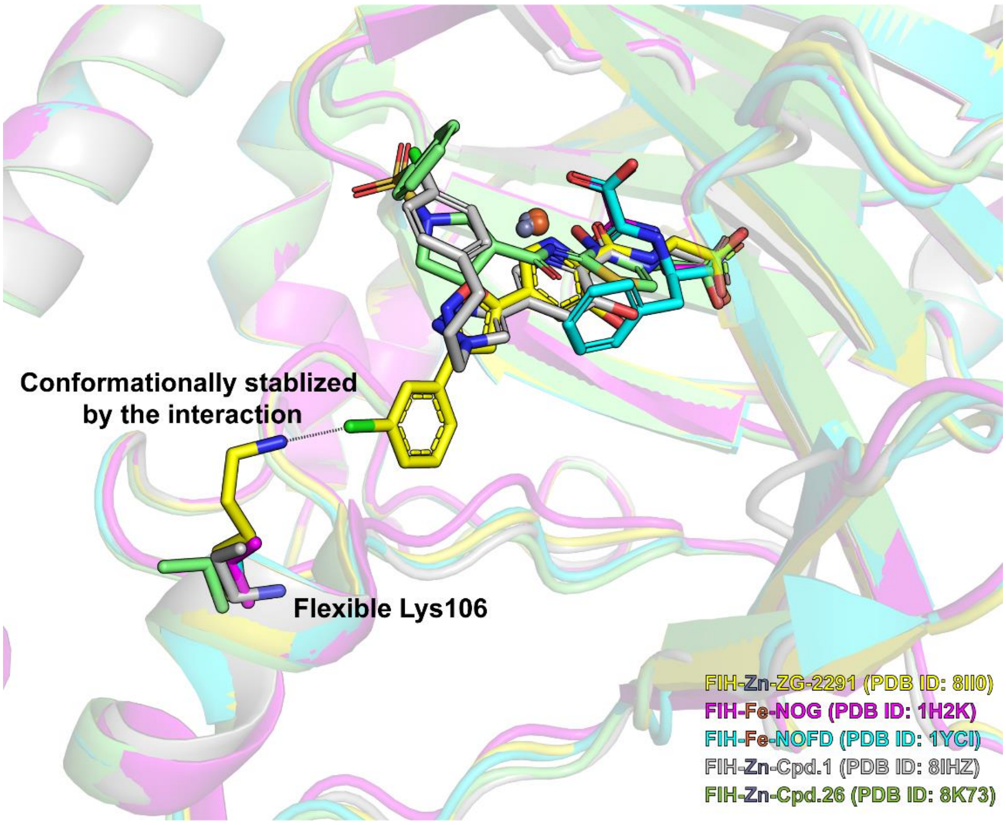

Figure S1. Superimposition of FIH crystal structure derived views analyzing the conformations of Lys106. Complexes shown: FIH in complex with **ZG-2291** (PDB ID: 8II0[1]) is shown in yellow; FIH in complex with NOG (PDB ID: 1H2K[2]) is shown in pink; FIH in complex with NOFD (PDB ID: 1YCI[3],) is shown in blue; FIH in complex with **Cpd.1** (PDB ID: 8IHZ[1]) is shown in grey; FIH in complex with **Cpd.26** (PDB ID: 8K73[4]) is shown in green. Note the *meta*-chloro substituent of **ZG-2291** is positioned to interact with the N^{ε} amino group of Lys106, for which clear electron density has been rarely observed in reported FIH structures.

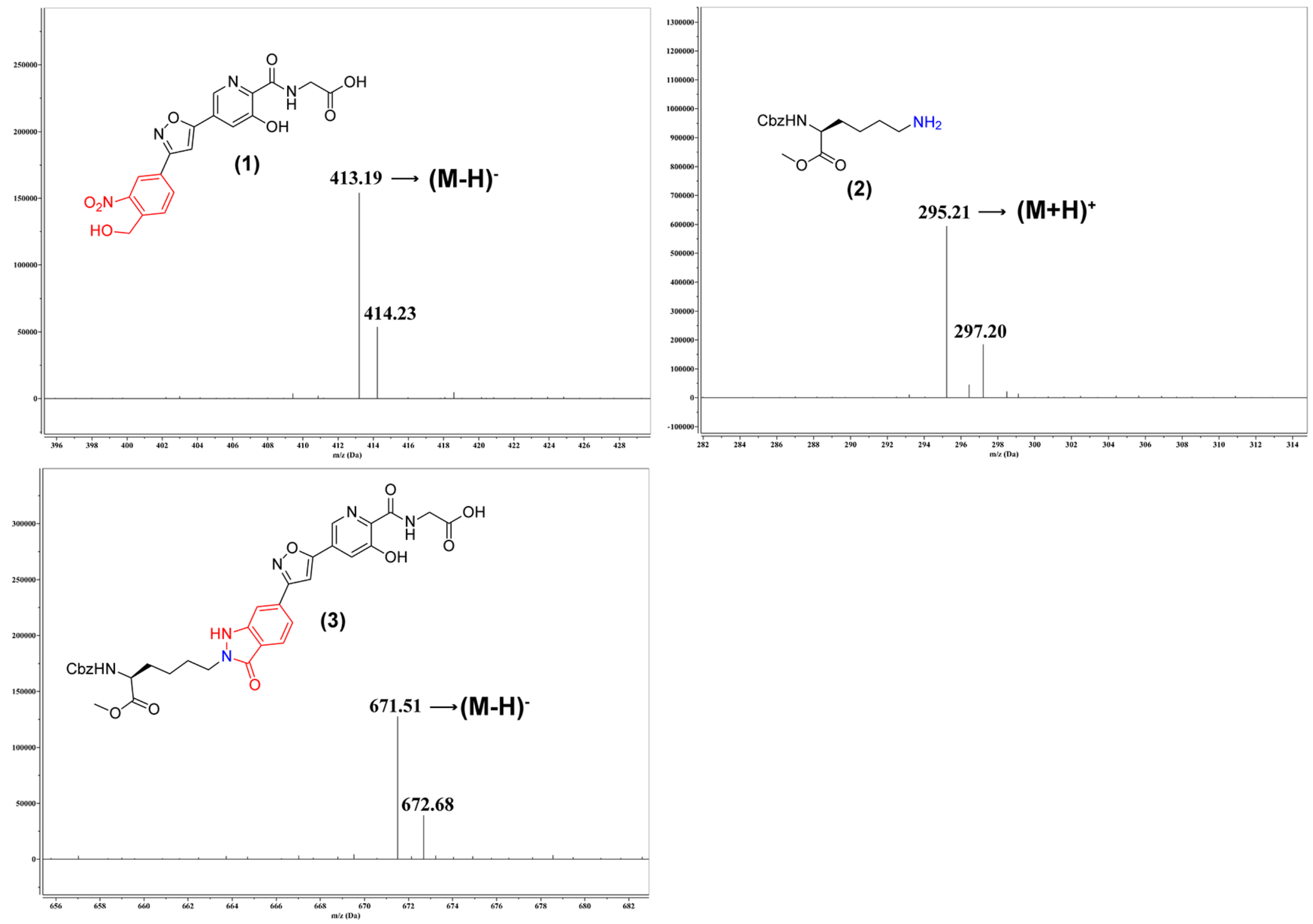

Figure S2. Mass spectrometry of **NBA-ZG-2291** (**1**), Cbz-Lys-OMe (**2**), and the cross-linked product (**3**). The mass spectrometry results for **NBA-ZG-2291** (**1**), Cbz-Lys-OMe (**2**), and the reaction product (**3**) confirm their identities. For each compound, the calculated mass (from the molecular formula) and the observed mass (from the spectra) are provided. **NBA-ZG-2291** (**1**): EI-MS: calcd. for $C_{18}H_{14}N_4O_8$ $[M-H]^-$ 413.07, found 413.19. Cbz-Lys-OMe (**2**): EI-MS: calcd. for $C_{15}H_{22}N_2O_4$ $[M+H]^+$ 295.17, found 295.21. Product (**3**): EI-MS: calcd. for $C_{33}H_{32}N_6O_{10}$ $[M-H]^-$671.21, found 671.51.

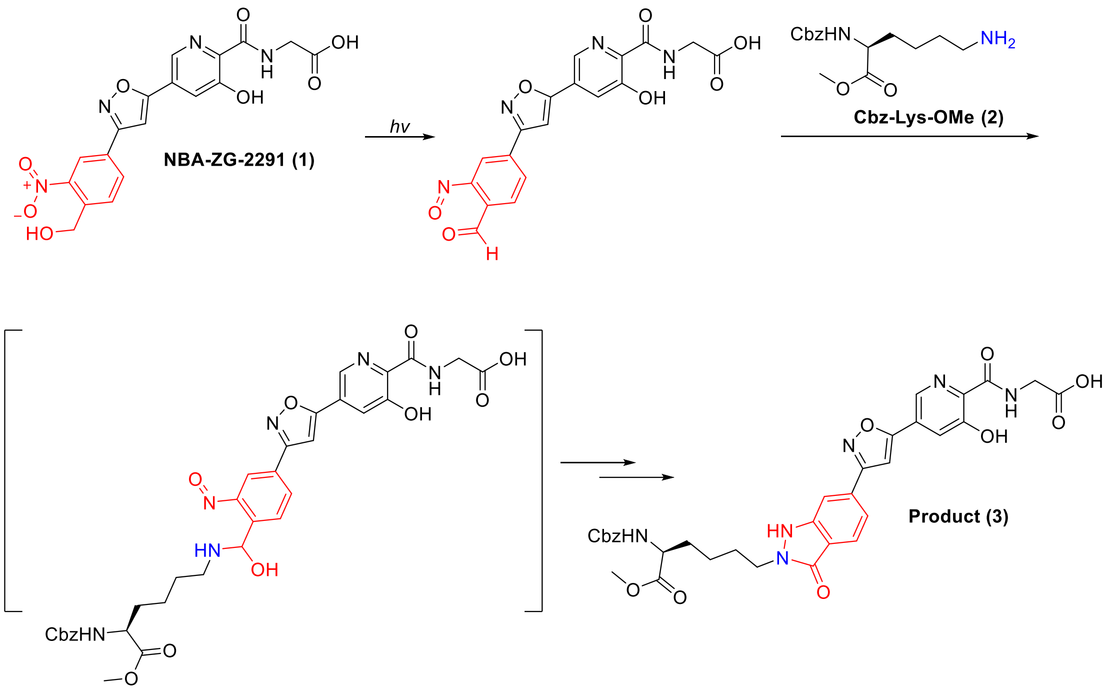

Figure S3. Proposed outline mechanism for the light-induced click reaction[5] between **NBA-ZG-2291** (**1**) and Cbz-Lys-OMe (**2**).

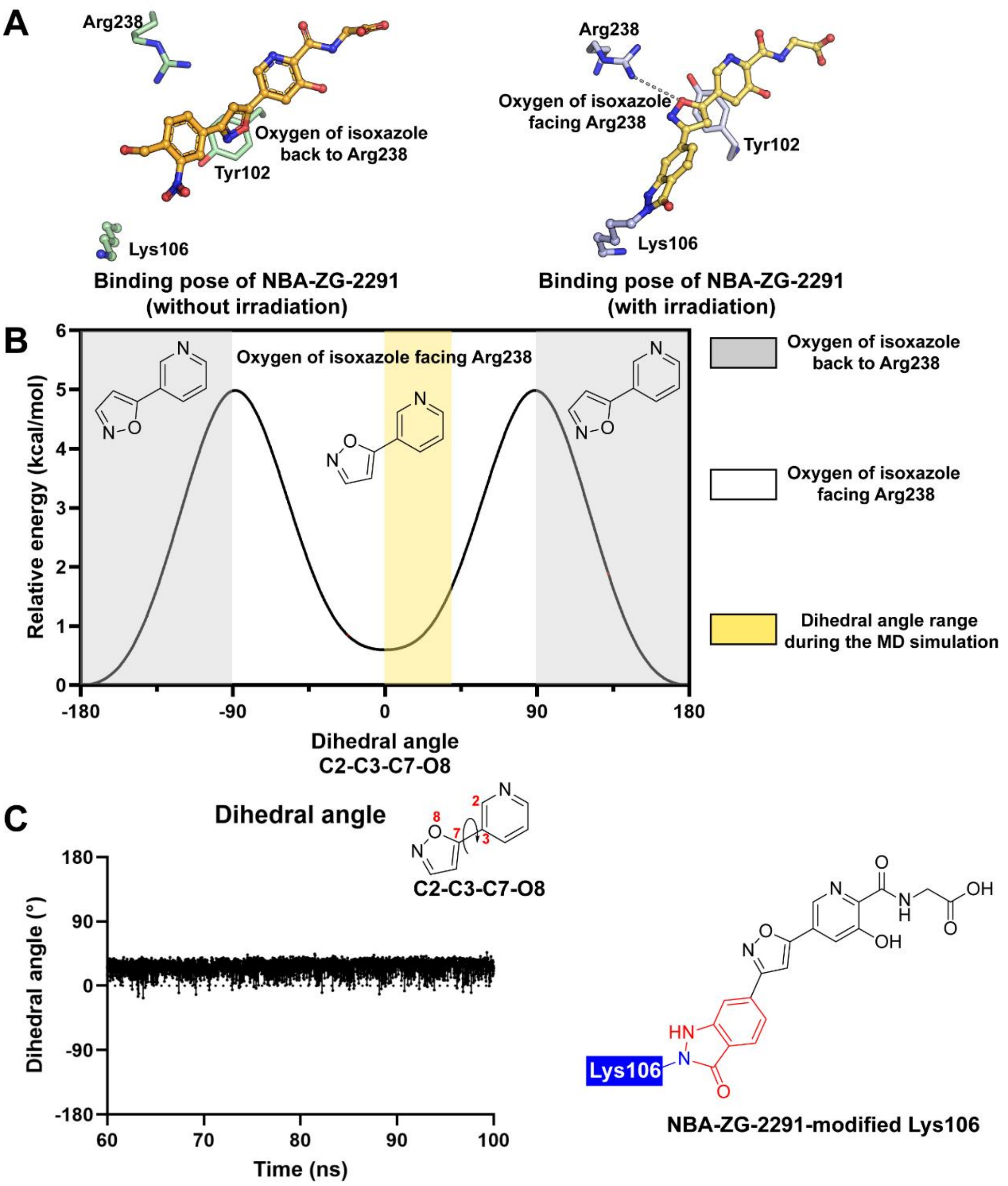

Figure S4. Dihedral angle analysis for the isoxazole-5-yl pyridine fragment. (A) Binding poses of **NBA-ZG-2291** to FIH without irradiation (as observed in a crystal structure) and with irradiation (as predicted pose by molecular dynamics simulations). (B) Dihedral angle scan by density functional theory (DFT) calculations for isoxazole-5-yl pyridine fragment of **NBA-ZG-2291**. The energy barrier associated with C3-C7 bond rotation is predicted to be <5 kcal/mol, indicating that rotation around this bond occurs freely. Note that the oxygen of isoxazole in the binding pose of **NBA-ZG-2291** faces towards Arg238 with which it is positioned to form a hydrogen bond. (C) From 60-80 ns, the dihedral angle of the isoxazole-5-yl pyridine fragment in **NBA-ZG-2291** converged to 30°, with the oxygen atom of the isoxazole facing Arg238.

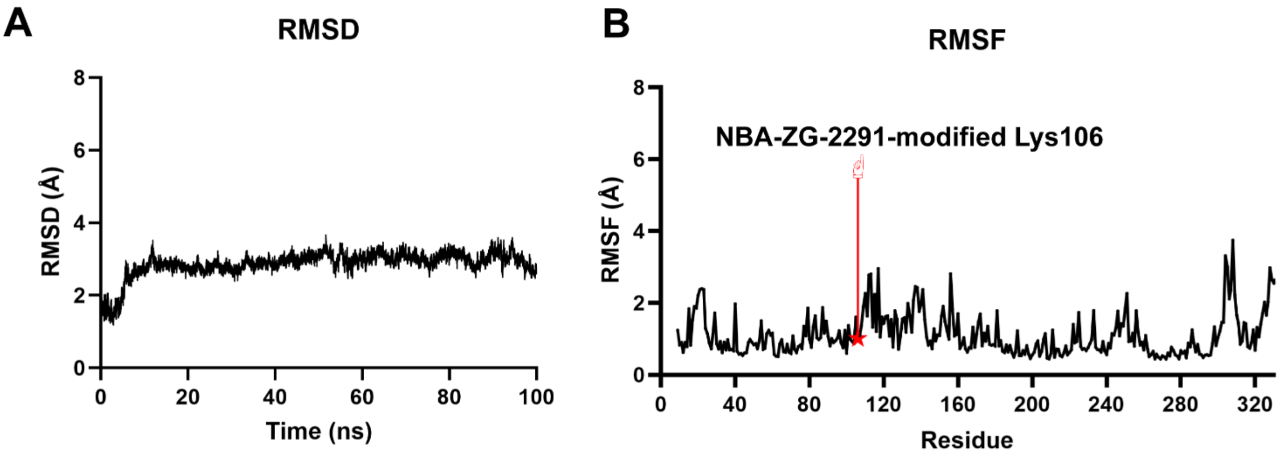

Figure S5. Molecular dynamics (MD) simulations of the **NBA-ZG-2291**-FIH complex. (A) The RMSD values of the FIH-**NBA-ZG-2291** complex rapidly stabilize at ~3 Å. (B) The RMSF value of Lys106 of FIH-**NBA-ZG-2291** complex was low, implying conformational stability of the Lys106 and **NBA-ZG-2291** covalently linked complex.

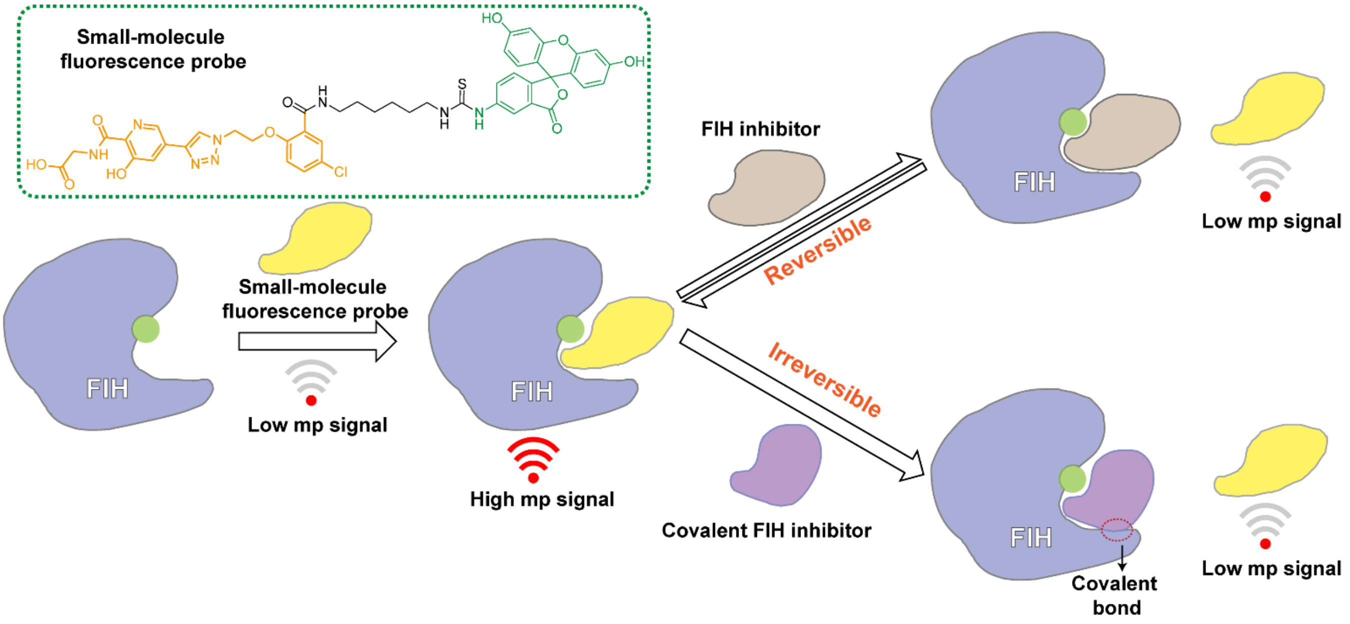

Figure S6. Schematic of the FP assay for FIH[1]. FP is used to investigate the binding of the probe and protein and displacement of the probe by reversible inhibitors and irreversible inhibitors. mP: milli polarization.

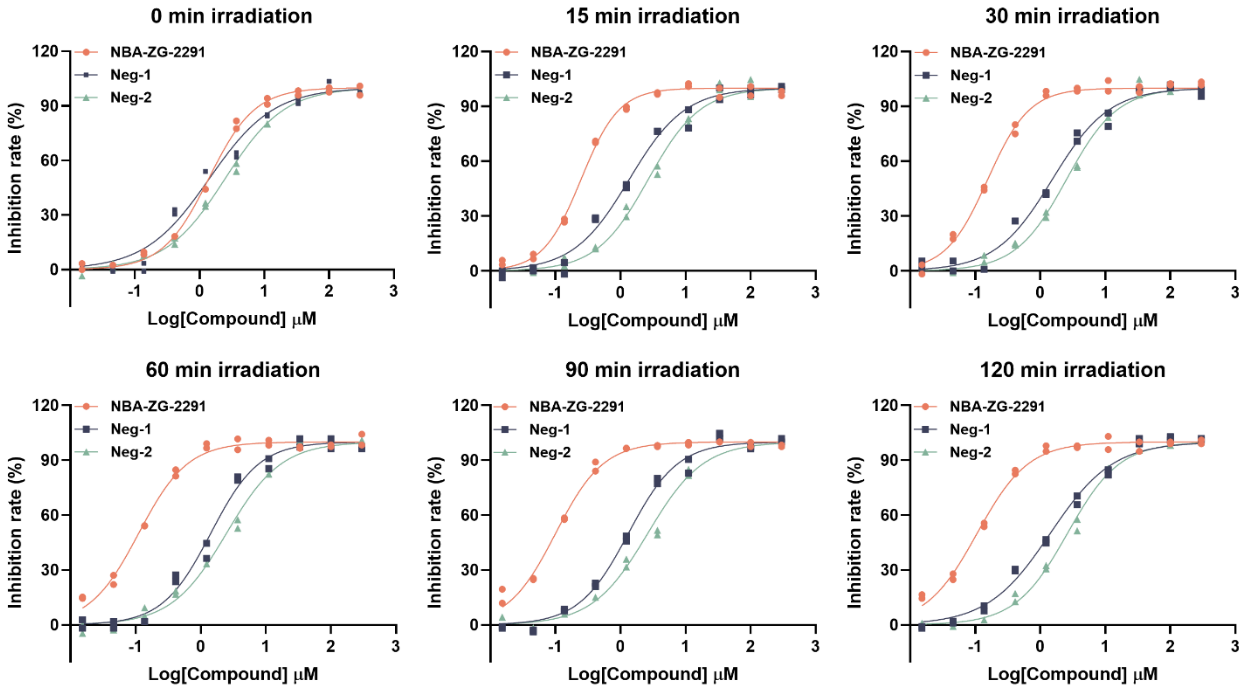

Figure S7. Inhibition curves for **NBA-ZG-2291**, **Neg-1**, and **Neg-2** against FIH^{WT} without and with light activation, as measured by the fluorescence polarization (FP) assay (Figure S6).[6] n = 2; mean ± SD.

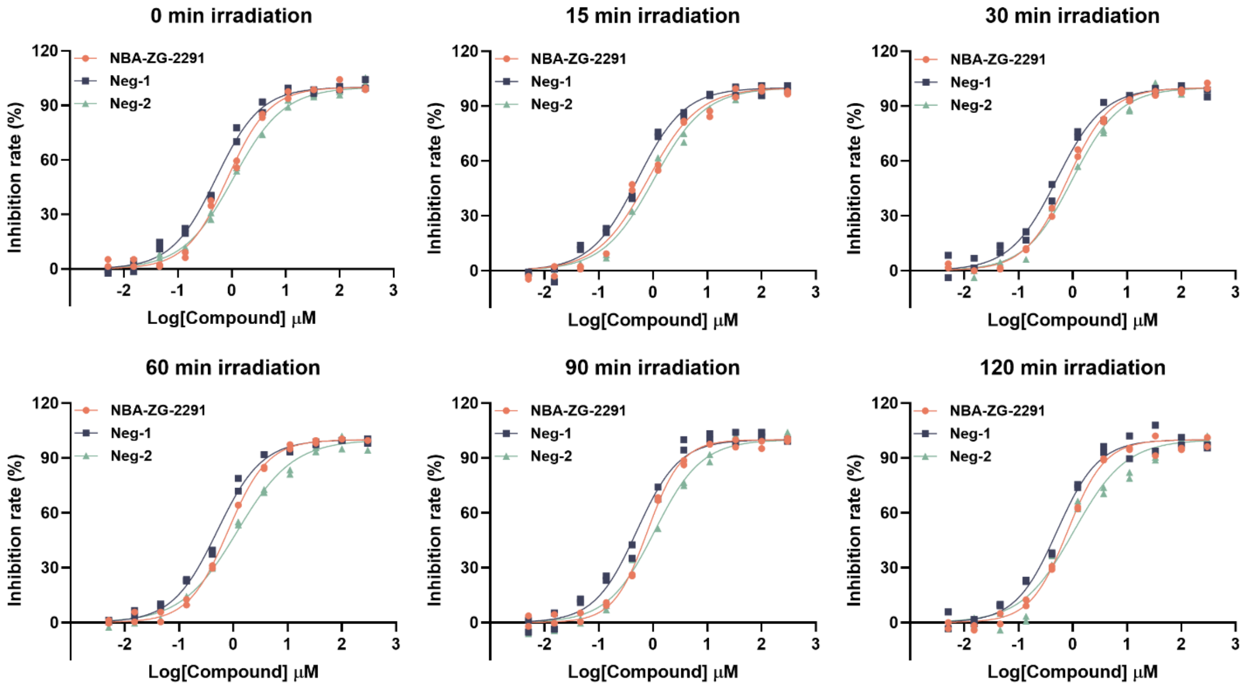

Figure S8. Inhibition curves of **NBA-ZG-2291**, **Neg-1**, and **Neg-2** against FIH^{K106A} without and with light activation, as measured by the fluorescence polarization (FP) assay (Figure S6).[6] n = 2; mean ± SD.

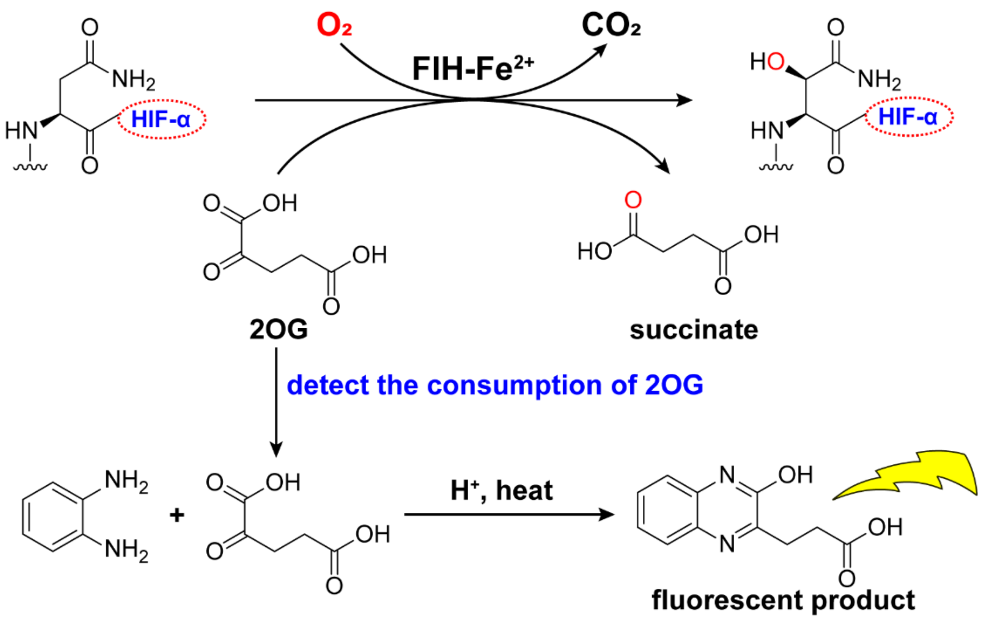

Figure S9. Schematic of the *O*-phenylenediamine (OPD) fluorescent derivative assay[6]. The assay monitors depletion of 2OG.

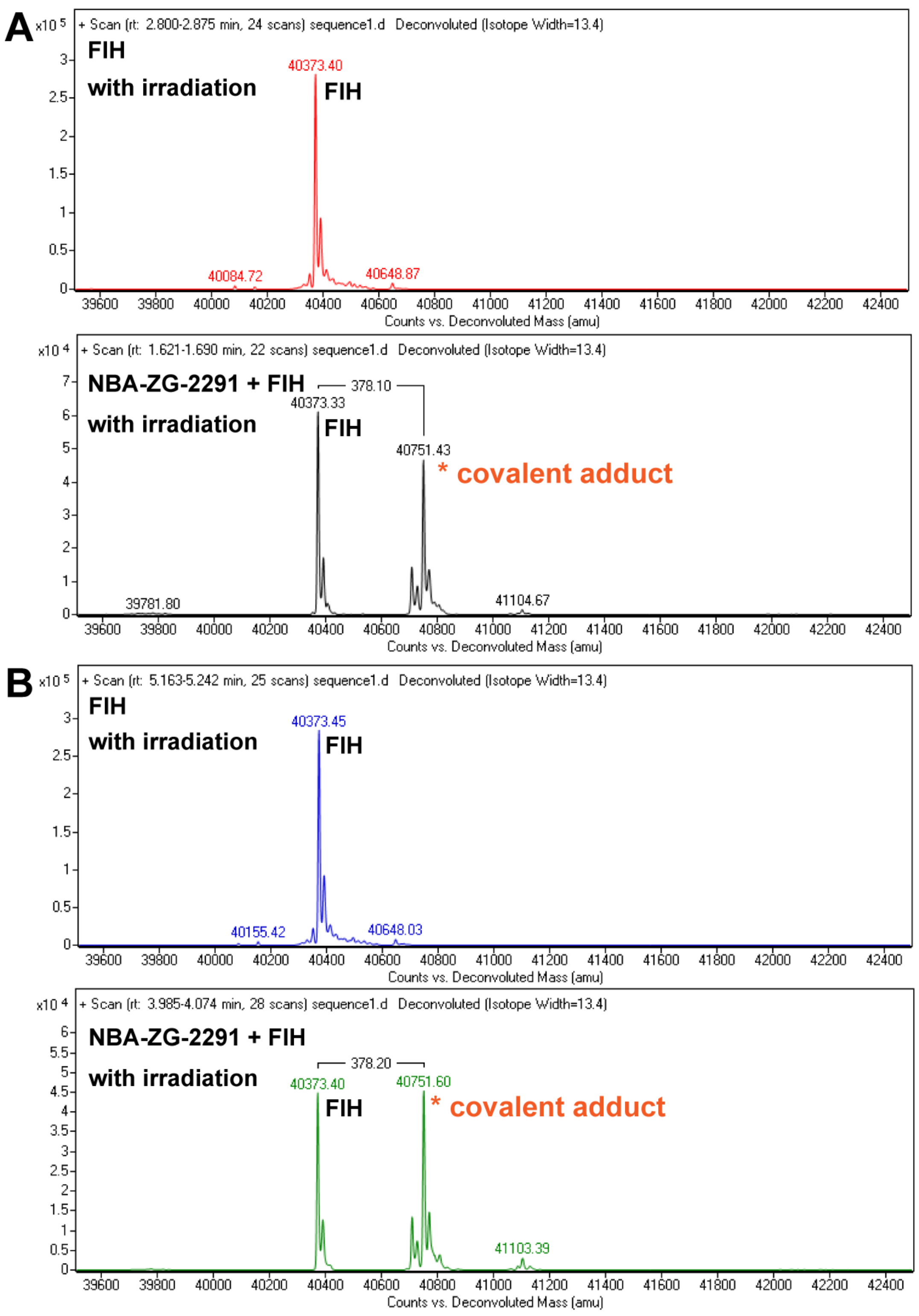

Figure S10. Replicates for intact protein mass analysis of FIH (5 µM) and FIH (5 µM) treated with **NBA-ZG-2291** (50 µM) with irradiation in Figure 3D. (A) First replicate. (B) Second replicate.

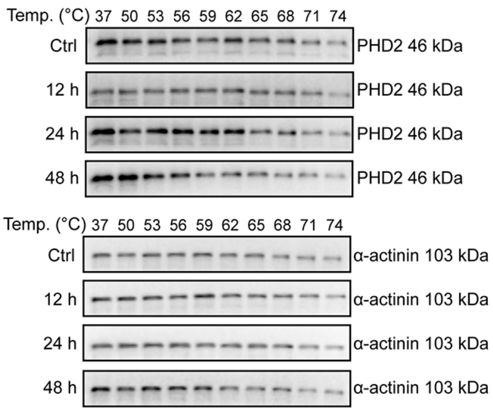

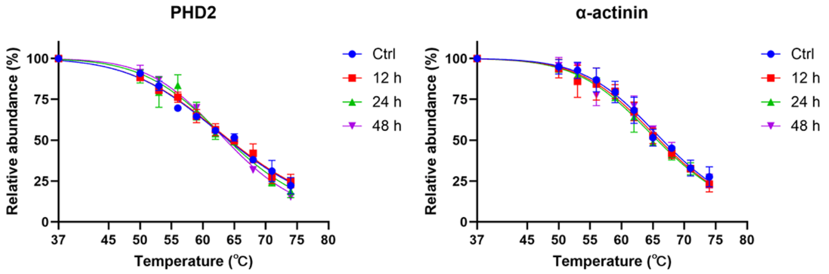

Incubation time	ΔT_m (°C)
Ctrl	-
12 h	0.22
24 h	-0.05
48 h	-0.41

Incubation time	ΔT_m (°C)
Ctrl	-
12 h	-0.48
24 h	-0.83
48 h	0.03

Figure S11. The CETSA assay detecting PHD2 engagement was carried out with Hep3B cells grown in the presence of **NBA-ZG-2291** (50 µM) for 12-48 h after 30 minutes of light activation (365 nm, 16W). The intensity of the bands for grayscale analysis was referenced to the starting temperature (37 °C). α-Actinin was the sample loading control. n = 2; mean ± SD. The uncropped blot is shown in Figure S14.

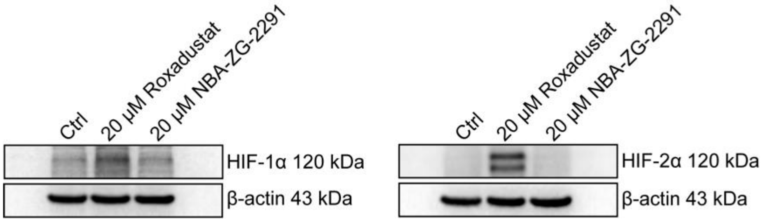

Figure S12. Representative western blot visualization of HIF-1α and HIF-2α levels in **NBA-ZG-2291** and Roxadustat-treated Hep3B cells; β-actin was used as the control for HIF-1α and HIF-2α loading. The uncropped blot is shown in Figure S14.

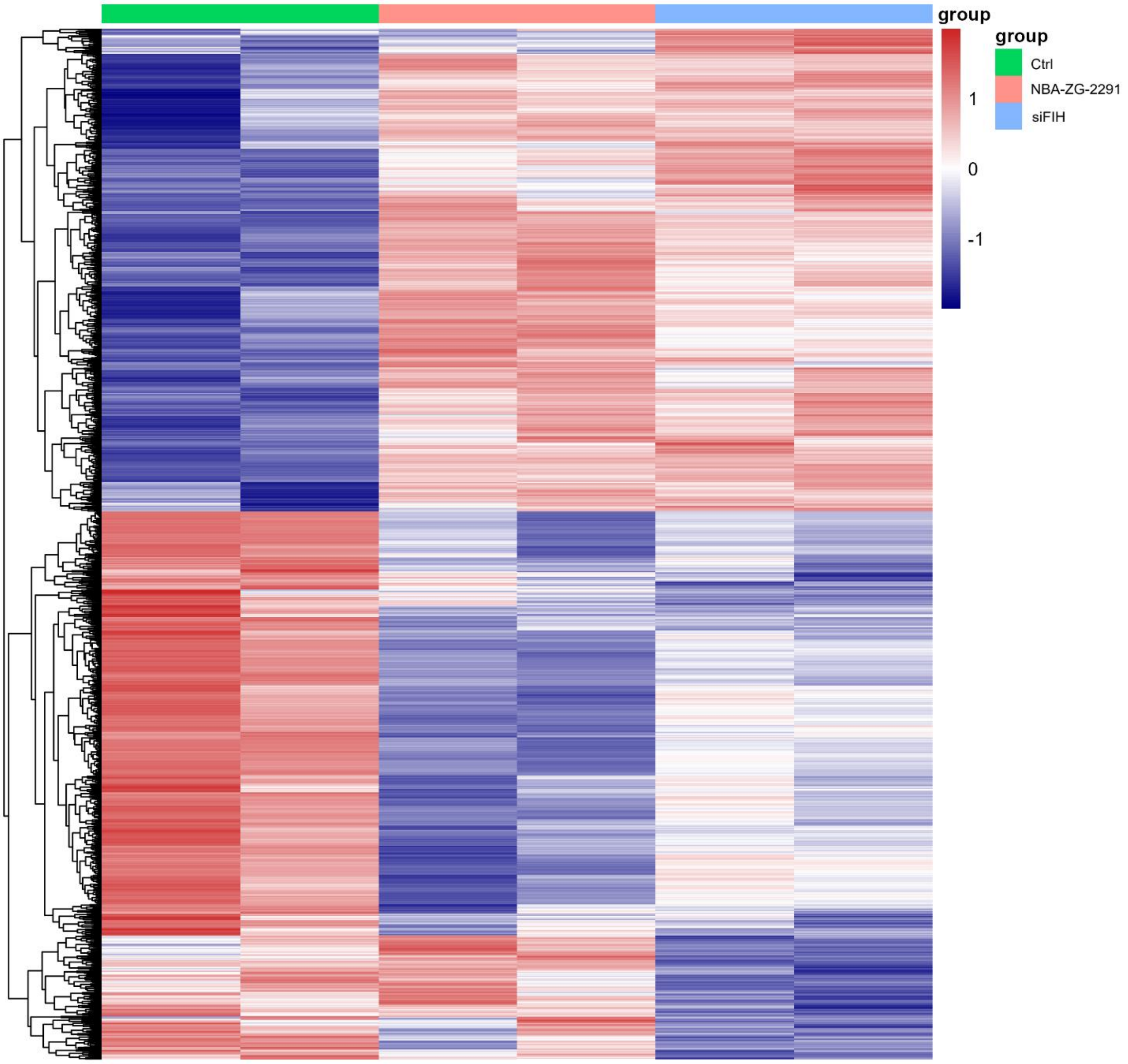

Figure S13. Clustering heatmap of differentially expressed genes (DEGs) for control, **NBA-ZG-2291**-treated, and siFIH groups.

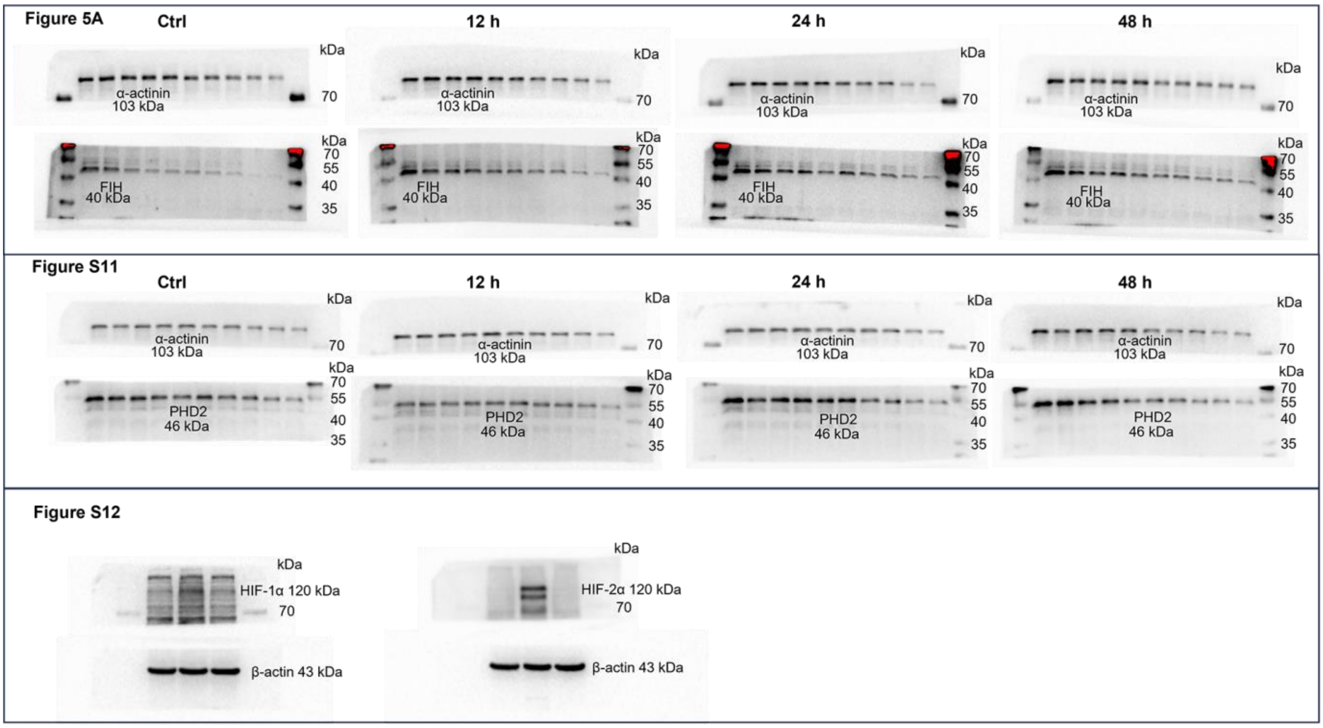

Figure S14. Uncropped images of all western blots in Figure 5A, Figure S11, and Figure S12.

2. Supplementary tables

Table S1. LC-MS Analysis of **Neg-1** and Cbz-Lys-OMe (**2**) Incubation in Hepes Buffer with Irradiation (365 nm, 16 W).

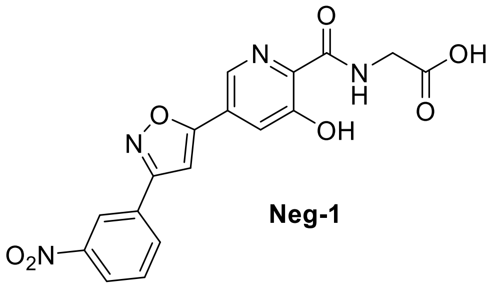

Neg-1

**Irradiation Time (h)**	**Peak area (Neg-1) t_R = 4.12 min**	**Peak area (2) t_R = 2.89 min**	**Peak area (product)**
**0**	1115815	94751	-
**1**	1111455	95024	-
**2**	1118094	94983	-
**3**	1113409	94563	-
**4**	1116789	95261	-

Table S2. LC-MS Analysis of **Neg-2** and Cbz-Lys-OMe (**2**) Incubation in Hepes Buffer with Irradiation (365 nm, 16 W).

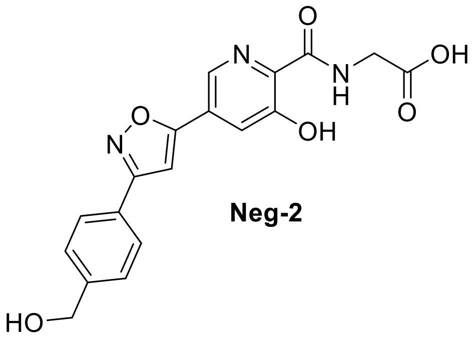

Neg-2

**Irradiation Time (h)**	**Peak area (Neg-2) t_R = 4.43 min**	**Peak area (2) t_R = 2.91 min**	**Peak area (product)**
**0**	909534	94443	-
**1**	910287	94905	-
**2**	904583	95231	-
**3**	911038	94894	-
**4**	911792	95117	-

Table S3. The Concentration of Remaining 2OG in the FIH Hydroxylation System after Incubation with **NBA-ZG-2291**.

**Concentration**	**Incubation Time**				
	**0 min**	**15 min**	**30 min**	**1 h**	**4 h**
**0 μM**	200.0	177.7	167.9	155.4	140.4
**0.14 μM**	200.0	187.5	184.6	174.8	163.1
**0.41 μM**	200.0	189.1	188.8	184.6	171.6
**1.23 μM**	200.0	191.6	190.6	187.9	182.8
**3.70 μM**	200.0	194.0	193.5	189.6	185.3
**11.1 μM**	200.0	199.7	198.7	197.8	197.2
**33.3 μM**	200.0	200.8	198.7	197.6	197.3
**100 μM**	200.0	200.1	199.2	198.5	198.3
**300 μM**	200.0	201.4	199.5	199.2	198.6

Table S4. The Concentration of Remaining 2OG in the FIH Hydroxylation System after Incubation with **ZG-2291**.

**Concentration**	**Incubation Time**				
	**0 min**	**15 min**	**30 min**	**1 h**	**4 h**
**0 μM**	200.0	177.7	167.9	155.4	140.4
**0.14 μM**	200.0	182.9	173.3	163.0	140.5
**0.41 μM**	200.0	184.2	176.4	162.3	153.7
**1.23 μM**	200.0	190.8	184.4	172.8	159.4
**3.70 μM**	200.0	192.8	190.8	181.0	167.9
**11.1 μM**	200.0	195.1	194.6	184.1	175.9
**33.3 μM**	200.0	195.6	195.3	189.4	181.4
**100 μM**	200.0	199.4	197.3	193.2	186.6
**300 μM**	200.0	200.0	199.1	196.6	193.3

Table S5. Data Collection and Refinement Statistics (Structures were Solved by Molecular Replacement).

	FIH·Mn^{II}·**NBA-ZG-2291**
**PDB ID**	9I4H
**Data collection**	
Space group	$P4_12_12$
Cell dimensions:	
a, *b*, *c* (Å)	a= 86.04, b= 86.04, c=147.40
α, β, γ (°)	α=90.0 β=90.0 γ=90.0
X-Ray source[b)]	I03 (DLS)
Resolution (Å)[c)]	60.84–2.30 (2.38-2.30)
R_{merge}	0.116 (2.084)
I / σI	18.0 (1.5)
CC (1/2)	0.999 (00.784)
Total number of reflections	669129 (62782)
Total number unique reflections	25421 (2433)
Completeness (%)	99.9 (99.4)
Multiplicity	26.3 (25.8)
**Refinement**	
R_{work} / R_{free}	0.204 / 0.218
No. atoms:	2871
B-factors:	71.89
R.m.s. deviations:	
Bond lengths (Å)	0.004
Bond angles (°)	0.695

[a)] Experimental details are specified in the Experimental procedures section; [b)] DLS: Diamond Light Source; [c)] Values in parentheses are for highest-resolution shell.

3. Experimental procedures

3.1 Fluorescence polarization (FP) assays[1] with FIH^{WT} and FIH^{K106A}

pET28a vectors encoding for full-length FIH with an *N*-terminal His_6 tag or for FIH (K106A) with an *N*-terminal His_6 tag were purchased from Nanjing Zoonbio Biotechnology. Both the FIH^{WT} and $FIHK^{106A}$ protein were expressed from the respective plasmid in *Escherichia coli* BL21 at 20 °C for 16 h. *E. coli* cells were lysed and the lysate were clarified by centrifugation. His6-tagged FIH^{WT} or $FIHK^{106A}$ was purified on a HisTrap affinity column (GE Healthcare) by elution with an imidazole gradient. The final purified proteins were stored in PBS buffer.

FP assays were carried out using 384-well plates (#3575, Corning); the polarization signal was measured using a SPARK Multi-Mode Microplate Reader (Tecan). A reported small-molecule fluorescence probe was used[7, 8]. The experiments were performed at pH 7.4 in a final volume of 60 μL and contained the 5 nM fluorescence probe, 50 nM FIH^{WT} or $FIHK^{106A}$, inhibitors at different concentrations, 10 mM Hepes, 150 mM NaCl, 0.05% (v/v) Tween-20, and less than 1% (v/v) DMSO. After incubating with or without irradiation at room temperature, the fluorescence polarization was measured from the top of the well with polarized filters and optical modules for fluorescein (λ_{ex} = 485 ± 25 nM, λ_{em} = 535 ± 25 nM). IC_{50}s were determined for duplicate measurements by non-linear least-squares analysis using GraphPad Prism 8.0. The K_i values of the inhibitors were calculated using a reported equation optimized from the Cheng-Prusoff equation[9].

$K_i = I_{50} / [1 + ([L]_{50} + [P]_0)/K_d]$

K_d values were determined using a constant concentration of probe and titrating with protein. $[I]_{50}$ denotes the concentration of the free inhibitor at 50% inhibition, $[L]_{50}$ is the concentration of the free labeled ligand at 50% inhibition, $[P]_0$ is the concentration of the free protein at 0% inhibition.

3.2 *O*-Phenylenediamine (OPD) fluorescent derivative assay

Assays were carried out by mixing 1 mM DTT, 0.6 mg/mL of catalase, 200 μM 2-OG, 50 mM Hepes, 200 μM HIF-1α (788-807), and inhibitors at pH 7.0 to a final volume of 44 μL and incubated at 37 °C for 5 min. At the same time, FIH enzyme and iron (prepared as 200 mM stock in 20 mM HCl and diluted with water) were mixed at room temperature for 3 min. Then, a 6 μL mixture of enzyme and iron was added to the 44 μL mixture above, and the reaction mixture was incubated at room temperature for 30 min. The reaction was stopped by the addition of 100 μL of 0.5 M HCl. After adding 50 μL of 10 mg/mL OPD in 0.5 M HCl and heating at 95 °C for 10 min, the derivatization was achieved. Then, 50 μL of the mixture centrifugated for 5 min was made basic by adding 30 μL of 1.25 M NaOH, and then fluorescence was detected by SPARK Multi-Mode Microplate Reader (Tecan).

3.3 X-ray crystallography

Crystallization experiments were carried out as reported[10]. *N*-Terminally His_6-tagged FIH (12 mg/mL, final concentration) was mixed with manganese (II) chloride (0.5 mM) in 50 mM Tris Buffer (pH 7.5) and incubated at 4°C for 5 min. Compound (2 mM) was added and the mixture was incubated at 4 °C for a further 15 min. The FIH-inhibitor mixture was then centrifuged with a MicroCL 21R (Thermo Fisher Scientific) at 14,000 rpm at 4°C for 10 min. Crystallization experiments were performed in 96-well, three-subwell, low profile Intelliplates (Art Robbins Instruments) using a Mosquito LCP (SPT Labtech) dispensing robot with 1.6 M ammonium sulfate, 6%w/v PEG400, and 0.1 M HEPES buffer (pH 7.5) as the precipitant solution. FIH crystals were grown using the sitting-drop vapor diffusion method at 20°C in 300 nL sitting drops with 2:1, 1:1, or 1:2 sample: precipitant solution ratios. Crystals were cryo-protected using mother liquor supplemented with 25%v/v glycerol before manual loop cryo-cooling in liquid N_2. Data for ligand-bound protein complexes were from single crystals at 100 K using Diamond Light Source (UK) beamlines. The FIH crystal structures were determined by molecular replacement (MR) using the AutoMR (PHASER) subroutine in PHENIX (version 1.18.2) based on the PDB ID: 1H2K crystal structure.

3.4 Intact protein mass spectrometry

Purified FIH (5 μM final) was incubated with **Lys-1** at 50 μM in 50 mM Tris pH 7.5, 50 μM $MnCl_2$ in a total volume of 100 μL. All experiments were incubated at 25 ℃ for 1 h, then with or without irradiation at 365 nm for 30 min. After the noted time, a 10 μL supernatant sample was separated using U3000 RSLCnano high performance liquid chromatography system. The mobile phase was a linear gradient of 5-95%$_{v/v}$ acetonitrile/water + 0.05% $_{v/v}$ formic acid. Assays were performed with the Orbitrap Eclipse mass spectrometry instrument. Data acquisition was carried out in Full MS mode, positive ion detection, the acquisition time was 10 min, and the scanning range of the mass-to-charge ratio was 400-2000 m/z.

3.5 Dihedral angle scan analysis

Dihedral angle scans were performed using the Gaussion 16 suite[11]. The original dihedral angles of the isoxazol-5-yl pyridine fragment were set to 0° via Gaussian View 6.0.16 for dihedral scanning from 0° to 360° in 1° increments at the B3LPY-D3(BJ)/6-31+G(d) level of theory. The resulting 360 conformations and the single-point energy for each conformation were extracted.

3.6 Molecular dynamics (MD) simulations

The process of customizing non-standard residues (**NBA-ZG-2291** modified Lys106) in the amber14sb force field involved the following steps: First, the molecular structure of **NBA-ZG-2291** was obtained and its topology was generated. Next, the PDB file (PDB ID: 8II0) was modified to meet amber14sb force field requirements, including adjusting atom names and adding hydrogen atoms. The molecular charges were then calculated using the RESP method, and the topology file was updated with these charge values. The RESP charges were fitted using Multiwfn, wherein the optimized structure and ESP data were inputted to perform the restrained electrostatic potential fitting. The obtained RESP charges were subsequently incorporated into the gaff topology generated by Multwfn. Finally, topology file's [atoms], [bonds], [angles], and other sections were edited to conform to amber14sb specifications, creating a customized residue file that can be used in simulations. Subsequently, MD simulations were performed via GROMACS (2019.06) software[12]. The system was solvated into a cube water box and then ionized with 0.15 M NaCl. After equilibration with the NVT ensemble for 1 ns and NPT ensemble for 1 ns, unrestrained MD simulations were undertaken for 100 ns. The representative conformations were extracted and mapped using PyMol 3.1 software.

3.7 Cell culture

Hep3B cells (SCSP-5045) were maintained in Minimum Essential Medium (MEM, GIBCO) with 10% fetal bovine serum (FBS, BI) and 100 U/mL penicillin and 100 μg/mL streptomycin (1 × P/S, NCM Biotech) in a 10% CO_2 and at 37 °C conditions.

3.8 Cellular thermal shift assay (CETSA)

Hep3B cells were treated with **NBA-ZG-2291** (50 μM) or DMSO for 12 h, 24 h, or 48 h. The cells were collected and washed with PBS buffer. Then, the cells were lysed by repeated freeze-thawing in liquid nitrogen for three cycles. The lysates were collected by centrifugation at 12000g for 30 min. The lysates were divided into equal volumes and heated at different temperatures for 3 min, then cooled at 4 °C The cell lysates were centrifuged at 4 °C; the supernatants were obtained for analysis by Western blotting. 10 μL lysate was loaded into a 12% SDS-PAGE gel and transferred onto a polyvinylidene difluoride membrane (pore size: 0.22 μm, Beyotime). Primary antibodies were incubated at 4 °C overnight; secondary antibodies were added onto the membrane with incubation at room temperature for 1 h. Immunoreactivities were detected with enhanced chemiluminescent autoradiography (Vazyme). Chemiluminescence was determined using Chemidoc XRS+ (Bio-Rad). The primary antibodies used in this study were: rabbit polyclonal anti-HIF1AN (1:1000, #10646-1-AP, proteintech), rabbit polyclonal anti-EGLN1 (1:1000, #19886-1-AP,

proteintech), mouse monoclonal anti-Alpha Actinin (1:2000, #66895-1-Ig, proteintech). The secondary antibodies used are goat anti-rabbit IgG-HRP (1:2000, #A0208, Beyotime) and goat anti-mouse IgG-HRP (1:5000, #sc-525409, santa Cruz Biotechnology).

3.9 Quantitative PCR analysis of gene expression

Total RNA was isolated from cells and tissues using Trizol regents (Vazyme). 1 μg RNA was used for reverse transcription using HiScript III RT SuperMix for qPCR (Vazyme). cDNAs were amplified in a ChamQ Universal SYBR qPCR Master Mix (Vazyme). Quantitative PCR was performed on ABI QuantStudio 3. PCR conditions: 3 min at 95 °C; 45 cycles of 10 s at 95 °C and 30 s at 60 °C; 15 s at 95 °C, 1 min at 60 °C and 15 s at 95 °C. The relative amount of mRNA was calculated after normalization to HPRT. Sequences for the primers are given as follows: *EGLN*-forward: 5'-CTGGTCCTCTACTGCGGGA-3', *EGLN*-reverse: 5'-AGCCACCATTGCCTTAGACCTC-3'; *HPRT*-forward: 5'-GACCAGTCAACAGGGGACAT-3', *HPRT*-reverse: 5'-AACACTTCGTGGGGTCCTTTTC-3'.

3.10 siRNA-mediated knockdown of FIH

Cells were seeded in 6-well plates at 50–60% confluency and transfected with FIH-specific siRNA (siFIH) or non-targeting control siRNA (siNC) using ExFect transfection reagent (Vazyme). In brief, siRNA (50 nM final concentration) and ExFect transfection reagent were mixed in Opti-MEM and incubated for 20 minutes at room temperature before being added to cells. Sequences for the siRNA are given as follows: FIH siRNA (siFIH): 5'-GACAAUCCCGACUACGAGATT -3' (sense) / 5'-UCUCGUAGUCGGGAUUGUCTT-3' (antisense); Non-targeting control siRNA (siNC): 5'-UUCUCCGAACGUGUCACGUTT-3' (sense) / 5'-ACGUGACACGUUCGGAGAATT-3' (antisense).

4. Synthesis

Materials were from commercial sources and were used as received. Organic solvents were concentrated via a rotary evaporator (EYELA OBS-2100) under reduced pressure (IKA VACSTAR digital) at 35-50 °C. Reactions were monitored using silica gel TLC plates (GF254, 0.25 mm) and visualized under UV (365/254 nm) light. A Mettler MP420 automatic melting point apparatus was used to determine melting points. Proton nuclear magnetic resonance (^{1}H NMR) and carbon nuclear magnetic resonance (^{13}C NMR) spectra were determined using Bruker AV-400/600 instruments using deuterated solvents with tetramethylsilane (TMS) as an internal standard. ^{1}H NMR chemical shifts (δ) are given in ppm ± 0.01 and coupling constants (J) are given in Hz ± 0.1 Hz. The spectra are reported as follows: δ/ppm (multiplicity, coupling constant(s) J/Hz, number of protons). Multiplicity is abbreviated as follows: s = singlet, d = doublet, dd = doublet of doublets, t = triplet, dt = doublet of triplet, q = quartet, dq = doublet of quartet, m = multiplet. ^{13}C NMR chemical shifts (δ) are given in ppm ± 0.01. High-resolution mass spectra (HRMS) were recorded on a Water Q-Tof micro mass spectrometer. The purity (≥95%) of the target compounds for biological testing was evaluated by the HPLC analysis using an Amethyst C18-P (4.6 × 150 mm, 5 μm, Waters) column eluting with methanol/water (90:10 v:v) with a flow rate of 0.5 mL/min; peaks were detected at 254 nm under UV.

Synthetic Procedures

Synthesis of A7

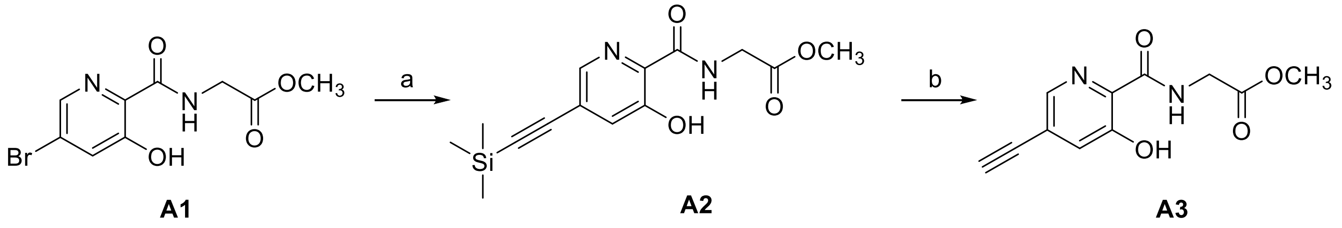

Scheme 1. *Reagents and conditions*: (a) Trimethylsilylacetylene, CuI, $Pd(PPh_3)_2Cl_2$, triethylamine, CH_3CN, reflux, 4 h, 74%; (b) TBAF, CH_3OH, rt, 1 h, 95%.

Methyl (3-hydroxy-5-((trimethylsilyl)ethynyl)picolinoyl)glycinate (**A2**)

To a solution of ingredient **A1** (2 g, 6.9 mmol) in CH_3CN (20 mL) were added trimethylsilicyne (0.86 g, 8.3 mmol), $Pd(PPh)_2Cl_2$ (0.97 g, 1.3 mmol), CuI (0.52 g, 2.7 mmol), and triethylamine (1 mL). The reaction mixture was heated to reflux for 4 h. After cooling to room temperature, the reaction mixture was filtered and the filtration was concentrated under reduced pressure to give the crude product, which was purified by column chromatography (eluent: petroleum ether/ ethyl acetate 8: 1) to afford **A2** as a white solid (1.60 g, 76%). mp: 115.3-117.1 °C (lit.[13] mp: 115.4-117.2 °C). ^{1}H NMR (300 MHz, Chloroform-*d*) δ 11.77 (s, 1H), 8.38 (s, 1H), 8.11 (d, J = 1.7 Hz, 1H), 7.35 (d, J = 1.7 Hz, 1H), 4.23 (d, J = 5.7 Hz, 2H), 3.81 (s, 3H), 0.27 (s, 9H). EI-MS: calcd. for $C_{14}H_{18}N_2O_4Si$ $[M+H]^+$ 307.1, found 307.1.

Methyl (5-ethynyl-3-hydroxypicolinoyl)glycinate (**A3**)

To a solution of **A3** (1.2 mg, 4.0 mmol) in CH_3OH (30 mL), TBAF (tetra-*N*-butylammonium fluoride) (1 mmol in THF, 4 mL, 4 mmol) was added slowly. The mixture was refluxed for 1 h, cooled to room temperature, then concentrated. The resulting residue was purified by column chromatography (eluent: petroleum ether/ethyl acetate (4:1)) to afford **A4** as a white solid (870 mg, 95%). mp: 133.5-134.9 °C (lit.[13] mp: 133.8-134.7 °C). ^{1}H NMR (300 MHz, Chloroform-*d*) δ 11.80 (s, 1H), 8.36 (s, 1H), 8.15 (d, J = 1.7 Hz, 1H), 7.39 (d, J = 1.7 Hz, 1H), 4.23 (d, J = 5.7 Hz, 2H), 3.81 (s, 3H), 3.48 (s, 1H). EI-MS: calcd. for $C_{11}H_{10}N_2O_4$ $[M+H]^+$ 235.1, found 235.1.

Synthesis of NBA-ZG-2291, Neg-1, and Neg-2

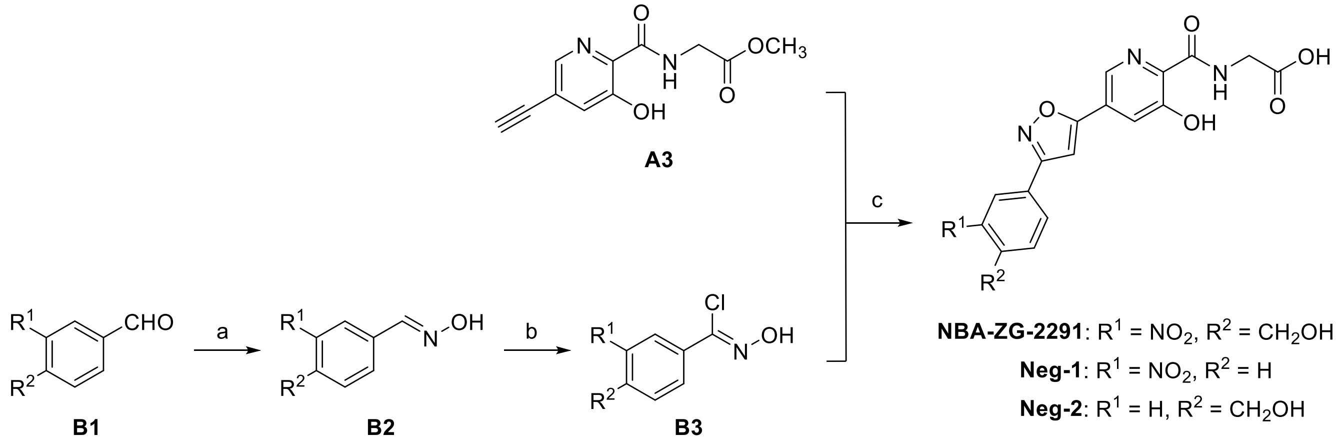

Scheme 2. *Reagents and conditions*: (a) NH_2OH•HCl, pyridine, EtOH, rt, 6 h; (b) NCS, DMF, rt, 16 h; (c) triethylamine, $CHCl_3$, 50 °C, 10 h and then 1 M LiOH, THF, rt, 1 h, 57-67% over two steps.

(3-Hydroxy-5-(3-(4-(hydroxymethyl)-3-nitrophenyl)isoxazol-5-yl)picolinoyl)glycine (**NBA-ZG-2291**) preparation via *N*-hydroxybenzimidoyl chlorides (**B3**)

To a solution of NH_2OH•HCl (1.30 g, 18.7 mmol) in EtOH (20 mL) were added pyridine (1 mL), substituted benzaldehyde (12.5 mmol). After stirring at room temperature for 6 h, 1 N hydrochloric acid (10 mL) was added slowly to the mixture. The mixture was extracted with ethyl acetate (30 mL×3). The combined organic layers were dried over anhydrous sodium sulfate and concentrated. The resulting residue (**B2**) was dissolved in DMF (15 mL) and then *N*-chlorosuccinimide (1.54 g, 11.5 mmol) was added slowly. After stirring at room temperature for 16 h, the mixture was poured into ice water (80 mL), then extracted with ethyl acetate (50

mL×3). The combined organic layers were dried over anhydrous sodium sulfate, filtrated, then concentrated to afford **B3**, which was used directly for the next step without further purification.

To a solution of **A3** (200 mg, 0.86 mmol) in $CHCl_3$ (10 mL) were added *N*-hydroxybenzimidoyl chloride (**B3**) (158 mg, 1.02 mmol) and triethylamine (1 mL). The mixture was heated to 50 °C under a nitrogen atmosphere for 10 h. After cooling to room temperature, saturated sodium bicarbonate (10 mL) was added slowly to the reaction mixture. The mixture was extracted with CH_2Cl_2 (20 mL×3). The combined organic layers were dried over anhydrous sodium sulfate, filtrated, and concentrated under reduced pressure to give the crude product. The crude product was dissolved in THF (20 mL) and 1 M LiOH (2 mL) was added to the above solution. The mixture was stirred at room temperature for 1 h, and then filtered and concentrated to about 2 mL. The resulting residue was neutralized by diluted hydrochloric acid to pH = 5. The precipitation was filtered and dried to afford **NBA-ZG-2291** as a light yellow solid (82 mg, 23%). m.p.: 217.1-218.8 °C. ^{1}H NMR (400 MHz, DMSO-*d*$_6$) δ 9.52 (t, *J* = 6.2 Hz, 1H), 8.74 (d, *J* = 1.8 Hz, 1H), 8.54 (d, *J* = 1.8 Hz, 1H), 8.31 (dd, *J* = 8.1, 1.8 Hz, 1H), 8.11 (s, 1H), 8.05 (d, *J* = 8.2 Hz, 1H), 7.93 (d, *J* = 1.8 Hz, 1H), 5.73 (s, 1H), 4.91 (s, 2H), 4.02 (d, *J* = 6.1 Hz, 2H). ^{13}C NMR (75 MHz, DMSO-*d*$_6$) δ 170.85, 168.69, 167.07, 161.46, 157.46, 147.46, 141.19, 137.02, 132.39, 131.65, 129.79, 127.99, 127.63, 122.59, 122.32, 102.06, 60.38, 41.24. HRMS (ESI): calcd. for $C_{18}H_{14}N_4O_8$ $[M+H]^+$ 415.0085, found 415.0894. HPLC (80% methanol in water): t_R = 2.87 min, 97.2%.

(3-Hydroxy-5-(3-(3-nitrophenyl)isoxazol-5-yl)picolinoyl)glycine (**Neg-1**).

The preparation method for compound **Neg-1** was analogous to that used for **NBA-ZG-2291**. Yield 41%. Light yellow solid. m.p.: 276.3-277.8 °C. ^{1}H NMR (300 MHz, DMSO-*d*$_6$) δ 12.56 (s, 1H), 9.51 (t, *J* = 6.1 Hz, 1H), 8.73 (d, *J* = 1.8 Hz, 1H), 8.67 (t, *J* = 1.9 Hz, 1H), 8.43 – 8.33 (m, 2H), 8.14 (s, 1H), 7.93 – 7.85 (m, 2H), 4.02 (d, *J* = 6.1 Hz, 2H). ^{13}C NMR (101 MHz, DMSO-*d*$_6$) δ 170.84, 168.76, 167.28, 161.69, 157.51, 148.77, 137.10, 133.13, 132.48, 131.52, 130.01, 127.67, 125.64, 122.46, 121.58, 102.27, 41.21. HRMS (ESI): calcd. for $C_{17}H_{12}N_4O_7$ $[M+H]^+$ 385.0779, found 385.0776. HPLC (80% methanol in water): t_R = 9.63 min, 96.9%.

(3-Hydroxy-5-(3-(4-(hydroxymethyl)phenyl)isoxazol-5-yl)picolinoyl)glycine (**Neg-2**).

The preparation method for compound **Neg-2** was analogous to that used for **NBA-ZG-2291**. Yield 22%. Light yellow solid. m.p.: 235.8-237.1 °C. ^{1}H NMR (300 MHz, DMSO-*d*$_6$) δ 12.57 (s, 1H), 9.51 (t, *J* = 6.2 Hz, 1H), 8.73 (d, *J* = 1.8 Hz, 1H), 7.95 – 7.86 (m, 4H), 7.51 (d, *J* = 8.0 Hz, 2H), 5.37 (s, 1H), 4.59 (s, 2H), 4.02 (d, *J* = 6.1 Hz, 2H). ^{13}C NMR (101 MHz, DMSO-*d*$_6$) δ 171.02, 168.42, 166.53, 163.13, 157.65, 145.71, 136.99, 132.49, 127.90, 127.46, 126.85, 122.39, 101.97, 62.93, 41.94. HRMS (ESI): calcd. for $C_{18}H_{15}N_3O_6$ $[M+H]^+$ 370.1034, found 370.1042. HPLC (80% methanol in water): t_R = 5.35 min, 96.6%.

References:

1. Wu, Y.; Chen, Y.; Corner, T.; Nakashima, Y.; Salah, E.; Li, Z.; Zhang, L.; Yang, L.; Tumber, A.; Sun, Z.; Wen, Y.; Zhong, A.; Yang, F.; Li, X.; Zhang, Z.; Schofield, C.; Zhang, X. A small-molecule inhibitor of factor inhibiting HIF binding to a tyrosine-flip pocket for the treatment of obesity. *Angew. Chem. Int. Ed.* **2024,** *63*, e202410438.

2. Elkins, J. M.; Hewitson, K. S.; McNeill, L. A.; Seibel, J. F.; Schlemminger, I.; Pugh, C. W.; Ratcliffe, P. J.; Schofield, C. J. Structure of factor-inhibiting hypoxia-inducible factor (HIF) reveals mechanism of oxidative modification of HIF-1 alpha. *J. Biol. Chem.* **2003,** *278*, 1802-1806.

3. McDonough, M. A.; McNeill, L. A.; Tilliet, M.; Papamicae¨l, C. A.; Chen, Q.; Banerji, B.; Hewitson, K. S.; Schofield, C. J. Selective inhibition of factor inhibiting hypoxia-inducible factor. *J. Am. Chem. Soc.* **2005**, 7680-7681.

4. Corner, T. P.; Teo, R. Z. R.; Wu, Y.; Salah, E.; Nakashima, Y.; Fiorini, G.; Tumber, A.; Brasnett, A.; Holt-Martyn, J. P.; Figg, W. D.; Zhang, X.; Brewitz, L.; Schofield, C. J. Structure-guided optimisation of *N*-hydroxythiazole-derived inhibitors of factor inhibiting hypoxia-inducible factor-α. *Chem. Sci.* **2023,** *14*, 12098-12120.

5. Zhu, J.; Kraemer, N.; Li, C.; Haddadin, M. J.; Kurth, M. J. Photochemical preparation of 1,2-dihydro-3*H*-indazol-3-ones in aqueous solvent at room temperature. *J. Org. Chem.* **2018,** *83*, 15493-15498.

6. McNeill, L. A.; Bethge, L.; Hewitson, K. S.; Schofield, C. J. A fluorescence-based assay for 2-oxoglutarate-dependent oxygenases. *Anal. Biochem.* **2005,** *336*, 125-131.
7. Li, Z.; Wu, Y.; Zhen, S.; Su, K.; Zhang, L.; Yang, F.; McDonough, M. A.; Schofield, C. J.; Zhang, X. In situ inhibitor synthesis and screening by fluorescence polarization: An efficient approach for accelerating drug discovery. *Angew. Chem. Int. Ed.* **2022,** *61*, e202211510.
8. Li, Z.; Zhen, S.; Su, K.; Tumber, A.; Yu, Q.; Dong, Y.; McDonough, M.; Schofield, C. J.; Zhang, X. A small-molecule probe for monitoring binding to prolyl hydroxylase domain 2 by fluorescence polarisation. *Chem. Commun.* **2020,** *56*, 14199-14202.
9. Nikolovska-Coleska, Z.; Wang, R.; Fang, X.; Pan, H.; Tomita, Y.; Li, P.; Roller, P. P.; Krajewski, K.; Saito, N. G.; Stuckey, J. A.; Wang, S. Development and optimization of a binding assay for the XIAP BIR3 domain using fluorescence polarization. *Anal. Biochem.* **2004,** *332*, 261-273.
10. Yeh, T. L.; Leissing, T. M.; Abboud, M. I.; Thinnes, C. C.; Atasoylu, O.; Holt-Martyn, J. P.; Zhang, D.; Tumber, A.; Lippl, K.; Lohans, C. T.; Leung, I. K. H.; Morcrette, H.; Clifton, I. J.; Claridge, T. D. W.; Kawamura, A.; Flashman, E.; Lu, X.; Ratcliffe, P. J.; Chowdhury, R.; Pugh, C. W.; Schofield, C. J., Molecular and cellular mechanisms of HIF prolyl hydroxylase inhibitors in clinical trials. *Chem. Sci.* **2017,** *8*, 7651-7668.
11. Frisch, M.J.; Trucks, G.W.; Schlegel, H.B.; Scuseria, G.E.; Robb, M.A.; Cheeseman, J.R.; Scalmani, G.; Barone, V.; Petersson, G.A.; Nakatsuji, H.; Li, X.; Caricato, M.; Marenich, A.; Bloino, J.; Janesko, B.G.; Gomperts, R., Mennucci, B.; Hratchian, H.P.; Ortiz, J.V.; Izmaylov, A.F.; Sonnenberg, J.L.; Williams-Young, D.; Ding, F.; Lipparini, F.; Egidi, F.; Goings, J.; Peng, B.; Petrone, A.; Henderson, T.; Ranasinghe, D.; Zakrzewski, V.G.; Gao, J.; Rega, N.; Zheng, G.; Liang, W.; Hada, M.; Ehara, M.; Toyota, K.; Fukuda, R.; Hasegawa, J.; Ishida, M.; Nakajima, T.; Honda, Y.; Kitao, O.; Nakai, H.; Vreven, T.; Throssell, K.; Montgomery, J.A.; Peralta, J.E.; Ogliaro, F.; Bearpark, M.; Heyd, J.J.; Brothers, E.; Kudin, K.N.; Staroverov, V.N.; Keith, T.; Kobayashi, R.; Normand, J.; Raghavachari, K.; Rendell, A.; Burant, J.C.; Iyengar, S.S.; Tomasi, J.; Cossi, M.; Millam, J.M.; Klene, M.; Adamo, C.; Cammi, R.; Ochterski, J.W.; Martin, R.L.; Morokuma, K.; Farkas, O.; Foresman, J.B.; Fox, D.J. Gaussian 16, Revision C.02, Gaussian, Inc., Wallingford CT, **2019**.
12. Abraham, M. J.; Murtola, T.; Schulz, R.; Páll, S.; Smith, J. C.; Hess, B.; Lindahl, E. GROMACS: High performance molecular simulations through multi-level parallelism from laptops to supercomputers. *SoftwareX* **2015**, *1-2*, 19−25.
13. Wu, Y.; Jiang, Z.; Li, Z.; Gu, J.; You, Q.; Zhang, X., Click chemistry-based discovery of [3-hydroxy-5-(1H-1,2,3-triazol-4-yl)picolinoyl]glycines as orally active hypoxia-inducing factor prolyl hydroxylase inhibitors with favorable safety profiles for the treatment of anemia. *J. Med. Chem.* **2018,** *61*, 5332-5349.

^{1}H NMR, HR-MS, and ^{13}C NMR spectra of A2-A3 and final products.

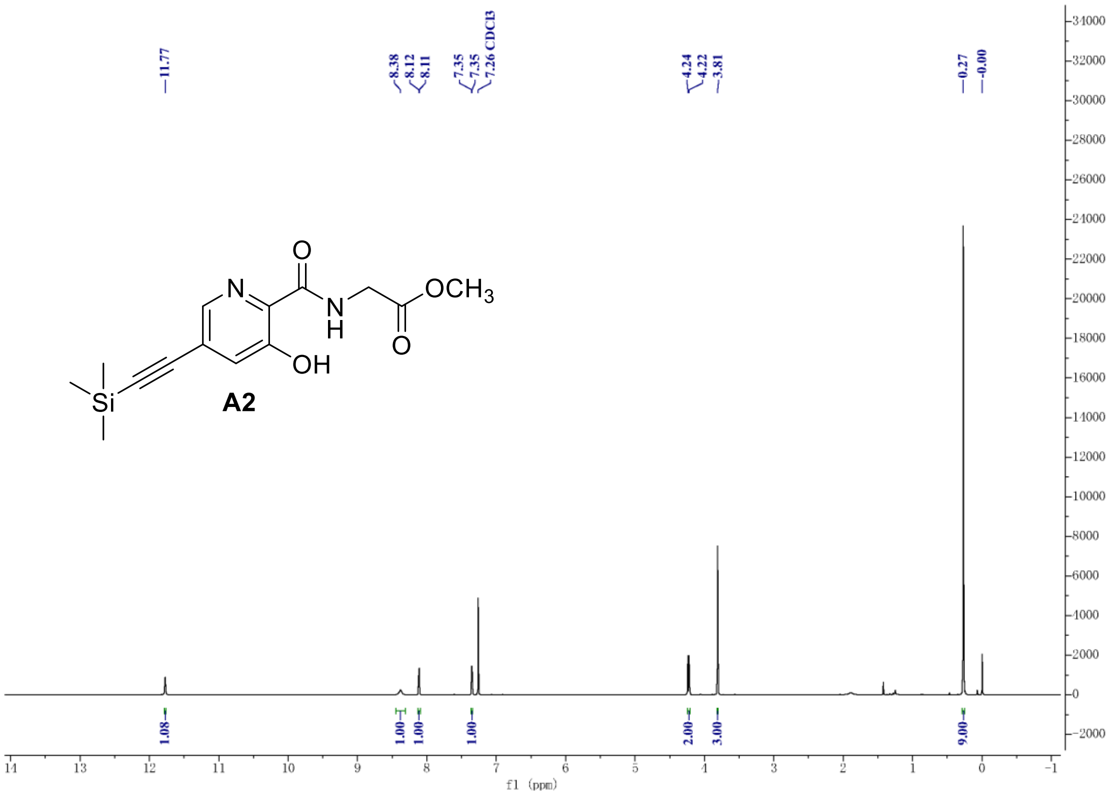

^{1}H NMR (300 MHz) of **A2** in DMSO-d_6.

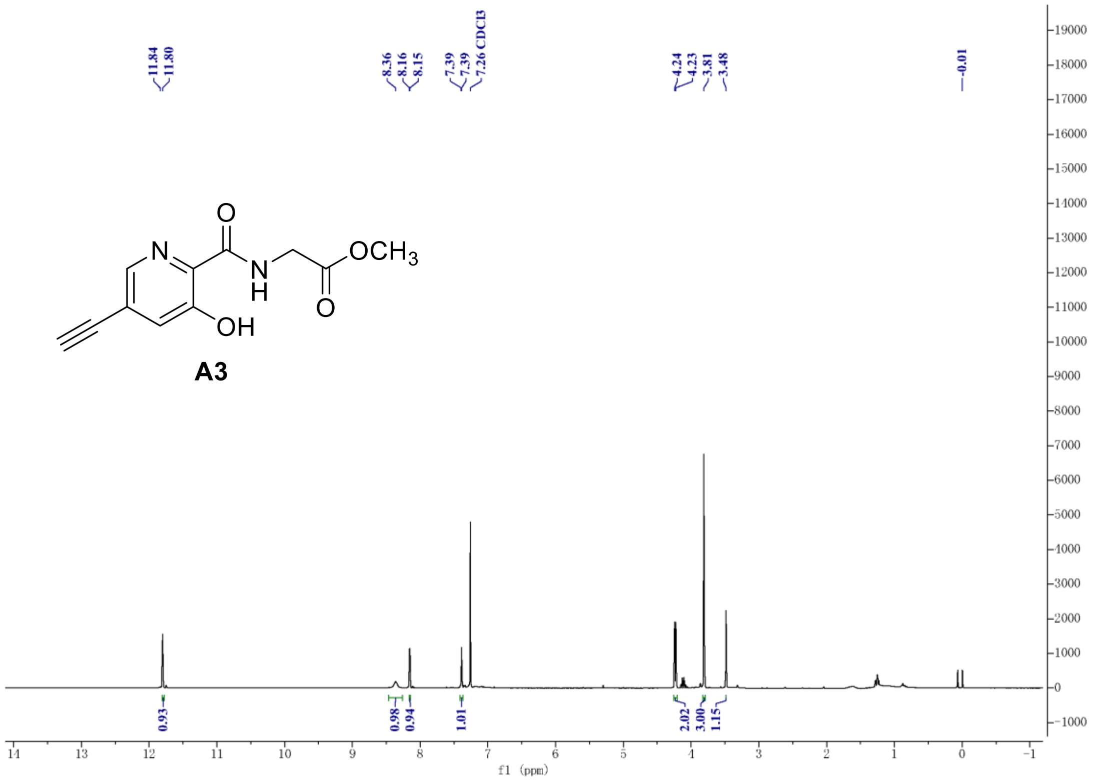

^{1}H NMR (300 MHz) of **A3** in DMSO-d_6.

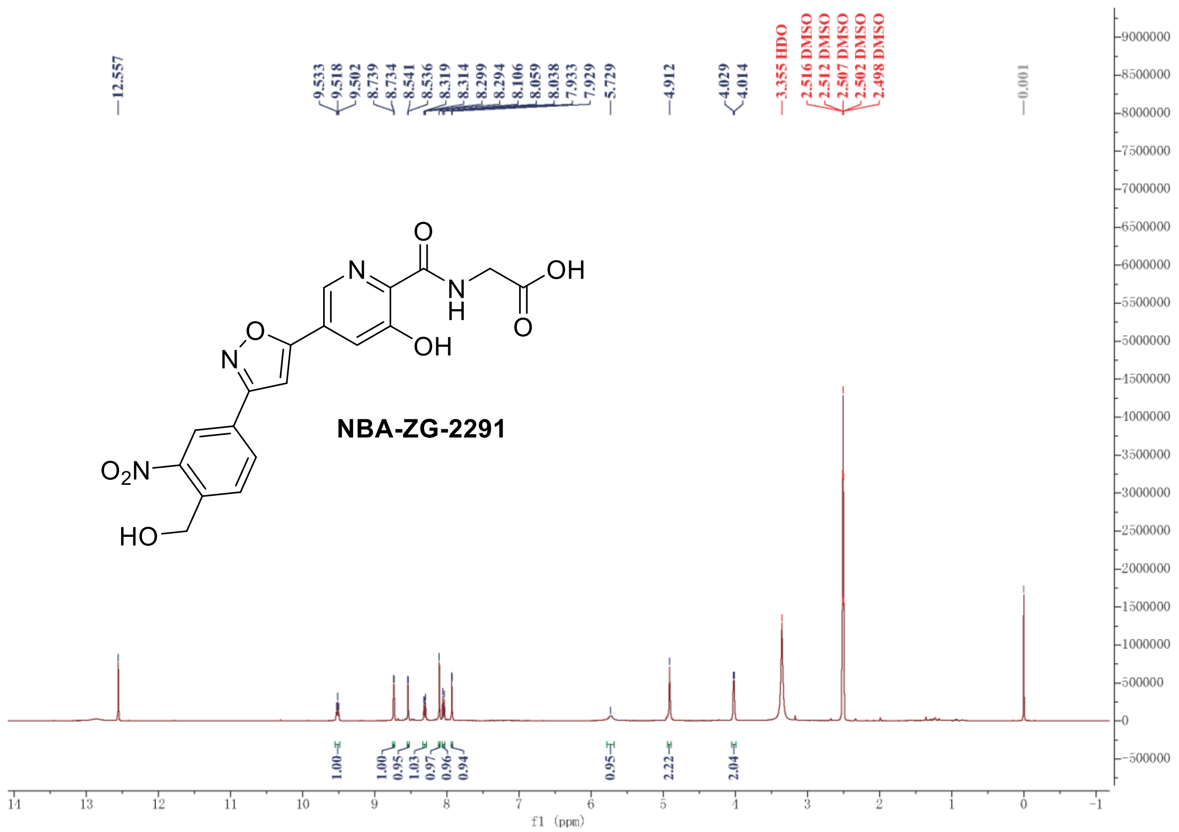

^{1}H NMR (400 MHz) of **NBA-ZG-2291** in DMSO-d_6.

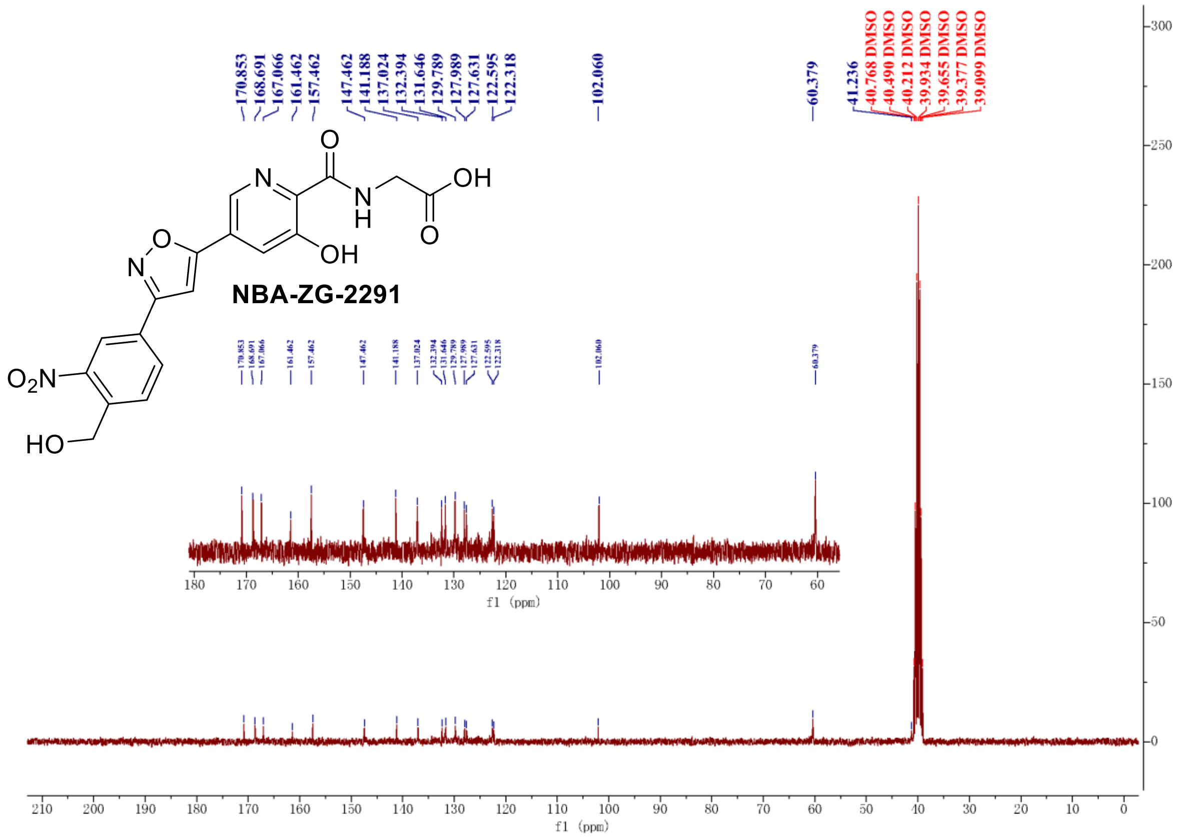

^{13}C NMR (75 MHz) of **NBA-ZG-2291** in DMSO-d_6.

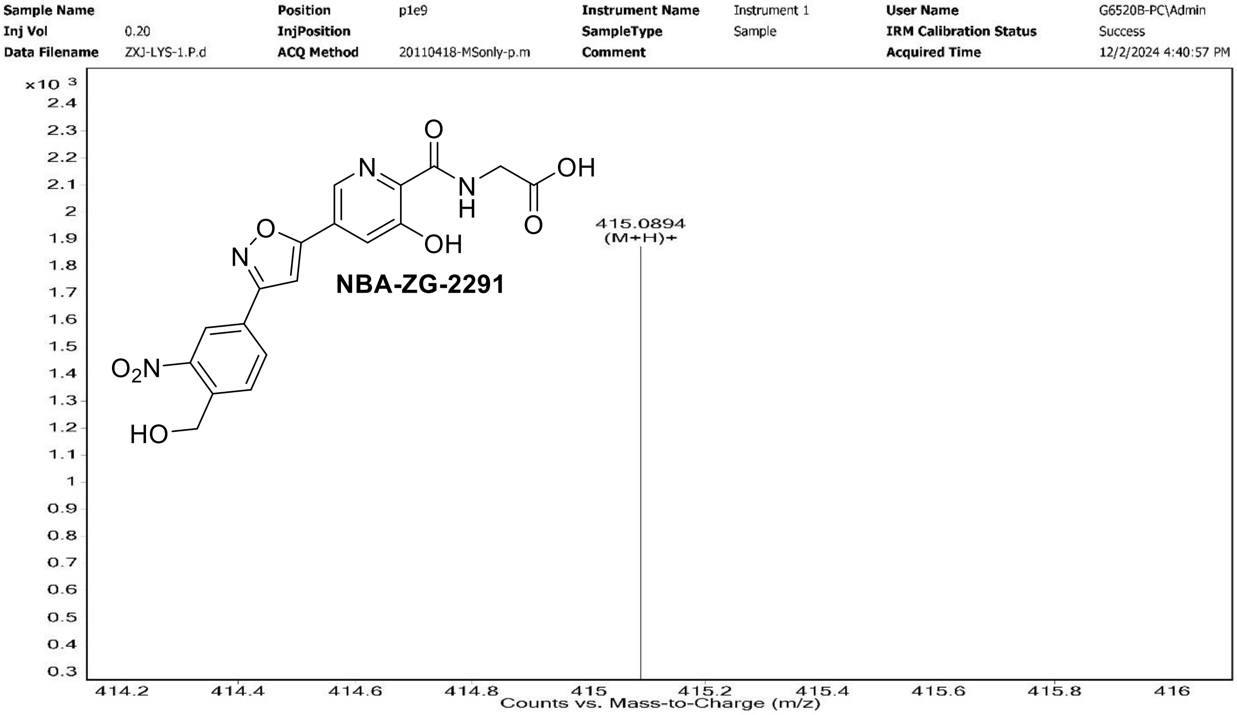

HRMS of **NBA-ZG-2291** in MeOH.

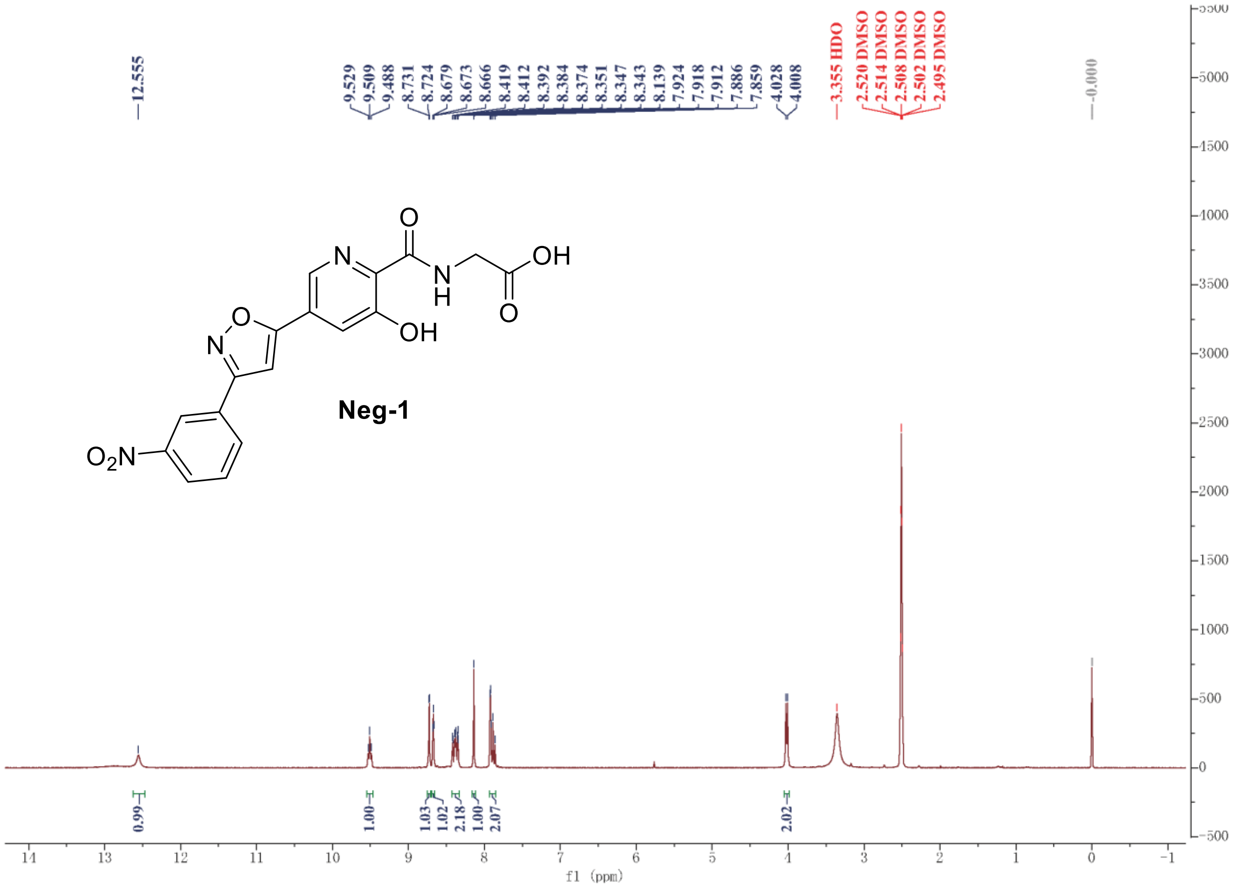

^{1}H NMR (400 MHz) of **Neg-1** in DMSO-d_6.

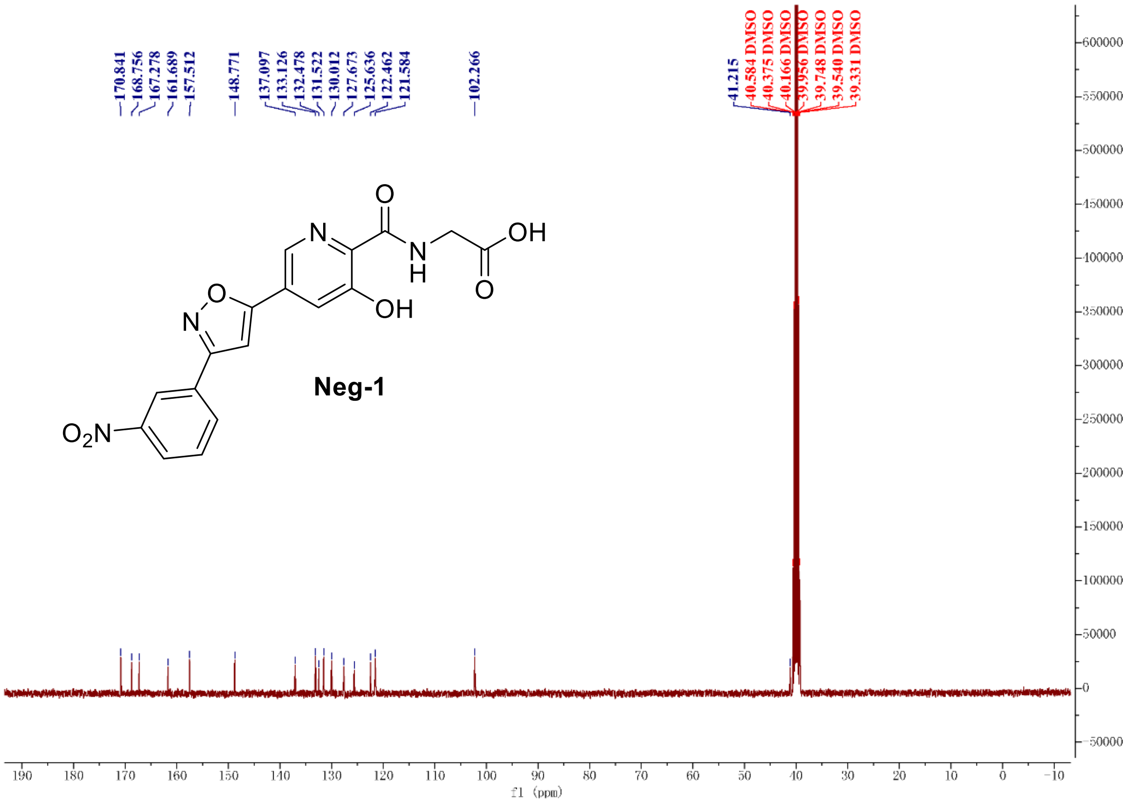

^{13}C NMR (101 MHz) of **Neg-1** in DMSO-d_6.

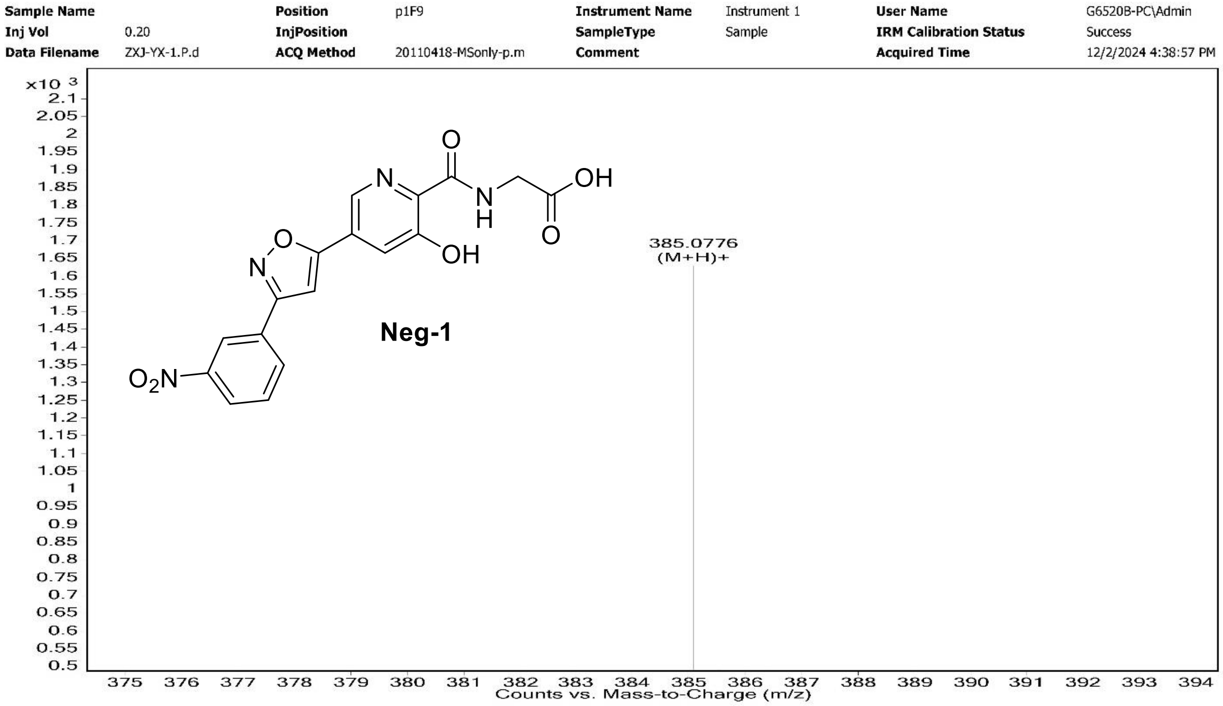

HRMS of **Neg-1** in MeOH.

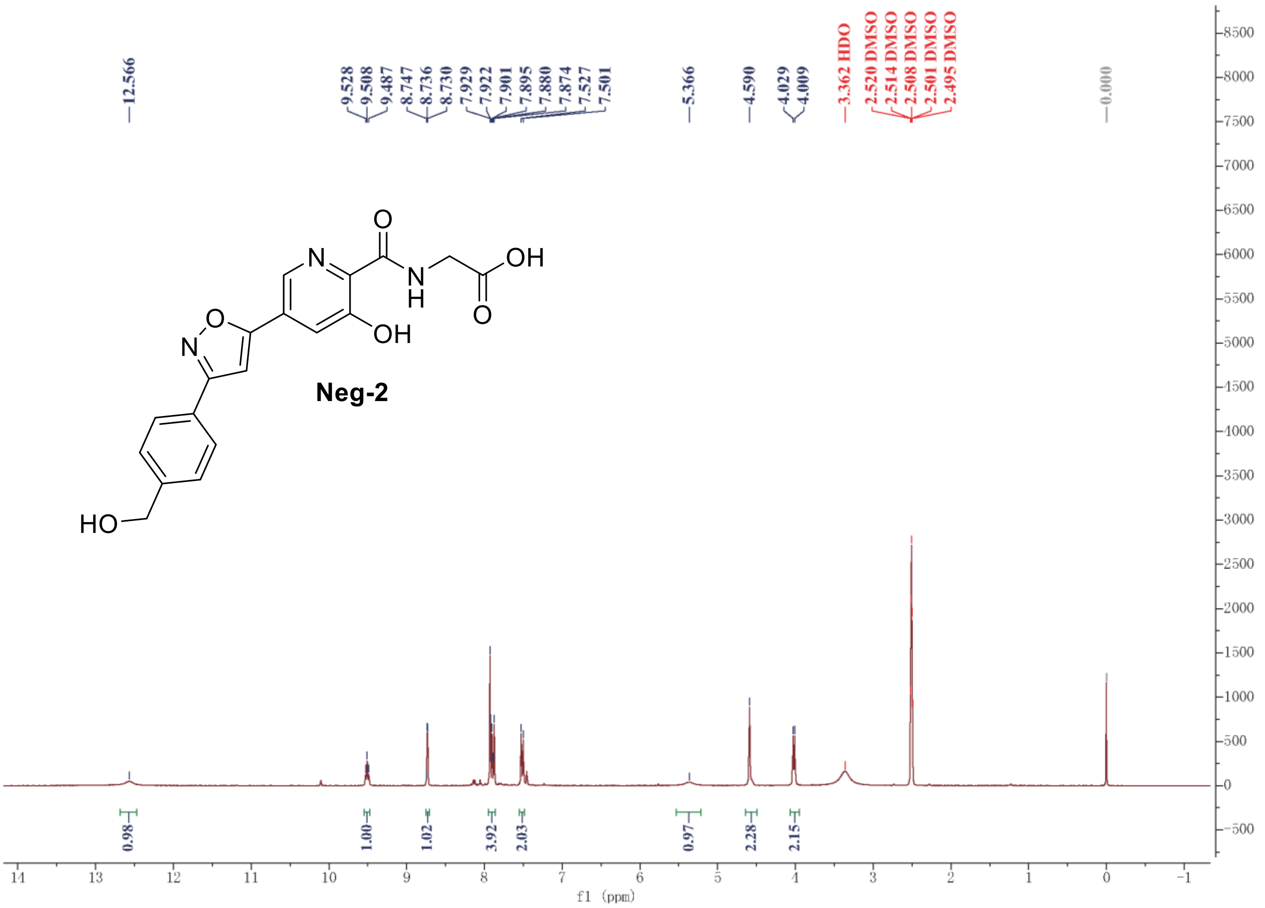

^{1}H NMR (400 MHz) of **Neg-2** in DMSO-d_6.

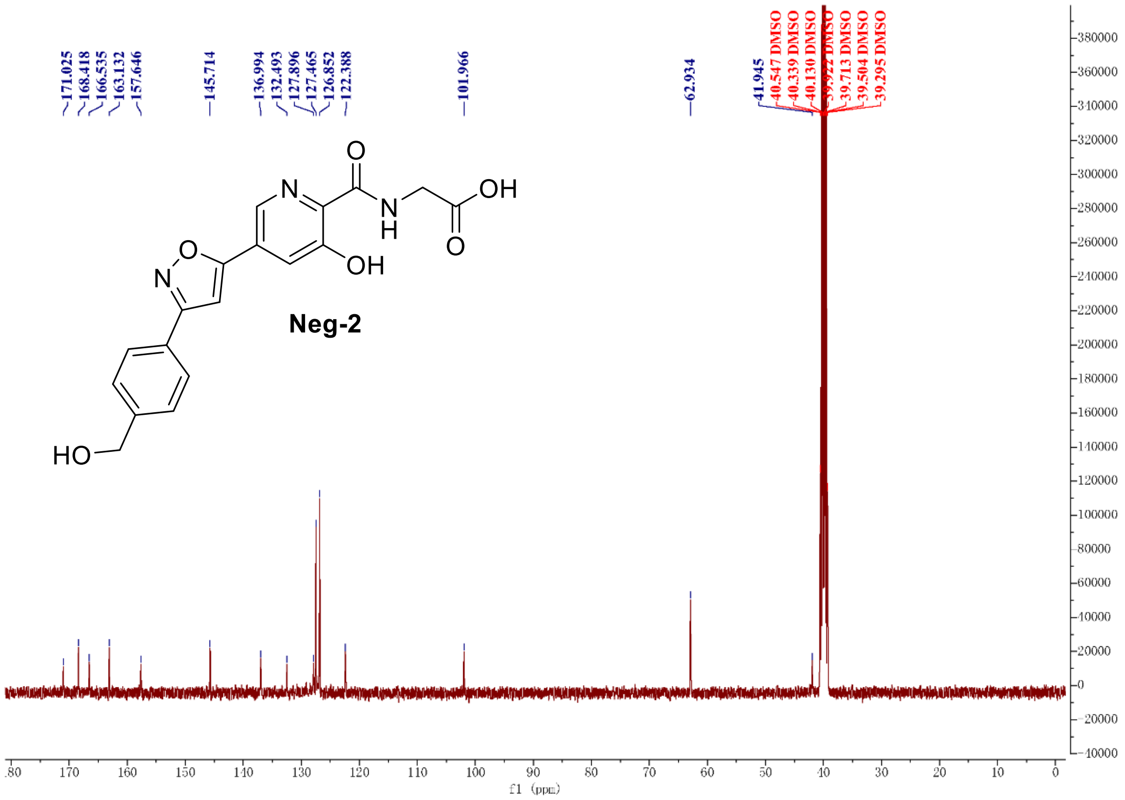

^{13}C NMR (101 MHz) of **Neg-2** in DMSO-d_6.

**Sample Name**		**Position**	p1e8	**Instrument Name**	Instrument 1	**User Name**	G6520B-PC\Admin
**Inj Vol**	0.20	**InjPosition**		**SampleType**	Sample	**IRM Calibration Status**	Success
**Data Filename**	ZXJ-YX-2.P.d	**ACQ Method**	20110418-MSonly-p.m	**Comment**		**Acquired Time**	12/2/2024 4:42:52 PM

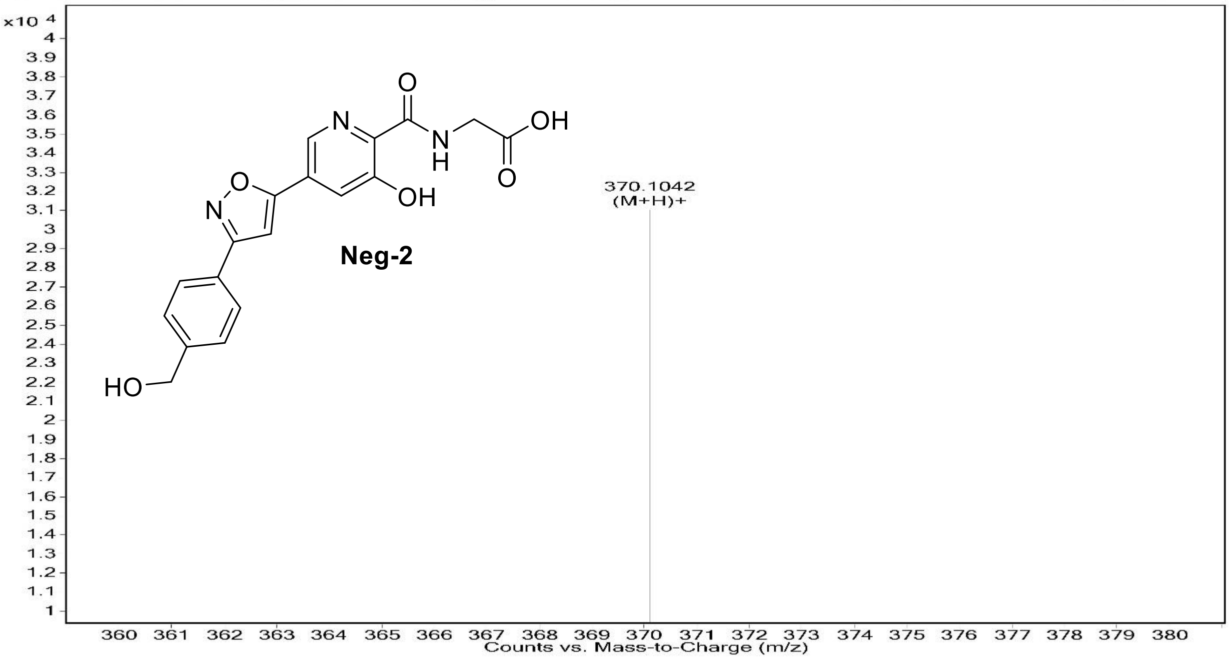

HRMS of **Neg-2** in MeOH.
